# A Healthy Brazil Nut Beverage with *Opuntia stricta* var. *dillenii* Green Extract: Beverage Stability and Changes in Bioactives and Antioxidant Activity during Cold Storage

**DOI:** 10.3390/foods13081237

**Published:** 2024-04-18

**Authors:** Daniel A. Alvarado-López, Sara Parralejo-Sanz, M. Gloria Lobo, M. Pilar Cano

**Affiliations:** 1 Laboratory of Phytochemistry and Plant Food Functionality, Biotechnology and Food Microbiology Department, Institute of Food Science Research (CIAL) (CSIC-UAM), Nicolás Cabrera 9, 28049 Madrid, Spain; darturlopez@hotmail.com (D.A.A.-L.);; 2 Department of Crop Production in Tropical and Subtropical Areas, Instituto Canario de Investigaciones Agrarias (ICIA), 38297 Santa Cruz de Tenerife, Spain; globo@icia.es

**Keywords:** Brazil nut, Brazil nut beverage, *Opuntia stricta* var. *dillenii* extracts, phenolic compounds, betalains, antioxidant activity, stability

## Abstract

Plant-based beverages are one of the foods that currently arouse a lot of interest in the population due to their composition with compounds beneficial to health in addition to their being used as milk substitutes for people who suffer from food disorders. Also, their fortification with different nutrients or healthy ingredients with the aim of improving plant-based health potential is actually gaining importance in the food industry. For this reason, the aim of the present investigation was the preparation of a healthy Brazil nut beverage enriched with *Opuntia stricta* var. *dillenii* pulp green extracts (ODPs), in order to produce a healthy plant-based beverage with improved nutritional characteristics. The microstructural characterization of the Brazil nut beverage, its stability during cold storage for up to 24 days at 5 °C, the composition of bioactive compounds provided via ODP extract (betalains and phenolic compounds), and their antioxidant activity were evaluated in this study. Green ODP extracts (0.5 and 1 g/100 g beverage) were added to a standardized Brazil nut beverage (reduced fat beverage). The characterization of the bioactive composition (betalains and phenolic compounds) of the elaborated beverage was achieved via HPLC (UV-vis and MS-QT of detection), and the antioxidant activity measurements via ORAC were also carried out. Optical microscopy, particle size, and Z potential analysis was conducted to characterize the structure of the Brazil nut beverages as food emulsions in which ODP extract was added. Most of the bioactive compounds from the green ODP extract added to the beverages showed good retention and remained stable throughout the 24 days of storage at 7 °C, with encapsulation efficiencies ranging from 98.34% to 92.35% for betalains and from 93.67% and 81.20% for phenolic compounds. According to the results of this study, Brazil nut beverage seems to be a healthy and efficient food emulsion system to encapsulate ODP extract rich in betalains and phenolic compounds, with high antioxidant activity, making possible the development of a Brazil nut beverage with improved health potential.

## 1. Introduction

Plant-based beverages could be considered aqueous extracts obtained from the decomposition of raw plant material. These beverages are commonly called vegetable or plant milks because they are usually taken as a substitute for cow’s milk, especially for people who have food restrictions such as lactose intolerance or an allergy to cow’s milk protein [1]. This makes their consumption very common because the incidence of allergy to cow’s milk is about 0.5% to 3.5% of people and lactose intolerance affects 65–75% of people [2]. However, there are many more reasons why plant-based beverages are consumed by many people, including a low content in saturated fats, cholesterol, and sugars, which helps prevent cardiovascular diseases and promotes weight loss, and the presence of bioactive compounds such as vitamins, phenolic compounds, tannins, and lignins, which are beneficial for health; moreover, their production is more environmentally friendly, and they are food beverages that are very attractive to vegetarians, vegans, and advocates of animal welfare [3].

In 2018, the plant-based beverage market experienced growth of 51.5%, which in economic terms represented 12.1 billion USD, in countries such as the United States; retail sales of plant-based milks increased by 20% in 2020, and it is estimated that 39% of American households consume plant-based beverages [4]. Regarding the European Union, Spain is the country where the most plant-based beverages are marketed and consumed, showing growth of 14% in 2018, and 24 million liters of plant-based beverages were sold in 2021; oat beverage, with growth of 25%, was the best seller, followed by soy and almond beverages [5].

There are different plant materials from which plant-based beverages can be obtained. Among the most used are cereals, grains, seeds, legumes, and nuts [6]. Of all these, nuts are usually one of the most used materials due to their high energy value because of their high content of unsaturated fat, large amounts of fiber, and minerals, as well as antioxidant compounds. This fact has led to a lot of research on their potential health benefits, among which a reduction in the risk of cardiovascular disease, a reduction in high cholesterol levels, and a reduction in the risk of diabetes stand out [7]. The nuts most studied and used for the production of plant-based beverages are almonds, hazelnuts, pistachios, and cashews [8]; however, there are other alternatives, such as the Brazil nut (*Bertholletia excelsa*), which is a type of Amazon nut that belongs to the *Lecythidaceae* family, in addition to Brazil. The Brazil nut is cultivated and marketed in Bolivia and Peru, but they are much less widespread than other types of nuts. Brazil nuts offer unique nutritional qualities compared to the other nuts because, in addition to having unsaturated fatty acids, dietary fiber, and phenolic compounds, they are also rich in selenium, which is a very important essential micronutrient due to its antioxidant capacity; it has been associated with health benefits such as a reduced risk of cancer and neurodegenerative diseases and the modulation of thyroid function [7].

**Brazil** nut (BN) beverage, as a plant-based drink, has the advantage of being able to incorporate certain ingredients to be fortified [4] because it essentially is a colloidal dispersion produced via the aqueous extraction of plant nut proteins, oil bodies, and biopolymers. BN showed an interesting nutritional quality related to its bioactive constituents (such as unsaturated fatty acids, phytosterols, tocopherols, squalene, phenolic compounds, proteins, and selenium). The high fat content of the Brazil nut (66.1%) leads to a high level of lipids in Brazil nut beverages (between 5 and 10%) [7] compared to other vegetable beverages such as almond, soybean, rice, or others (lipid content between 1 and 3.4%) [8]. In the present work, a partially defatted, standardized Brazil nut beverage (BNB) was developed for use as a food delivery system for bioactive compounds from *Opuntia stricta* var. *dillenii*. This plant-based beverage showed a nutritional composition of 2.9% total lipids, total proteins of 1.27%, and total carbohydrates of 1.06%, a profile which is within the range of most commercial vegetable milks, such as soybean and almond beverages [9]. Also, the Brazil nut beverage represented an important alternative source for obtaining the daily requirement of Se in the human diet. The hydrothermal processing in beverage production has a significant effect on the content of some minor lipid components (FFA, MG, total tocopherol, β-sitosterol, and squalene) via the hydrolysis of their triglycerides and certain conjugated compounds [8]. Together with these lipid components, the Brazil nut beverage also has an interesting composition of proteins, showing an higher band intensity above 20 kDa in electrophoretic analysis and suggesting a higher concentration of convicilin and 11S globulin protein [8].

**Figure 1:**
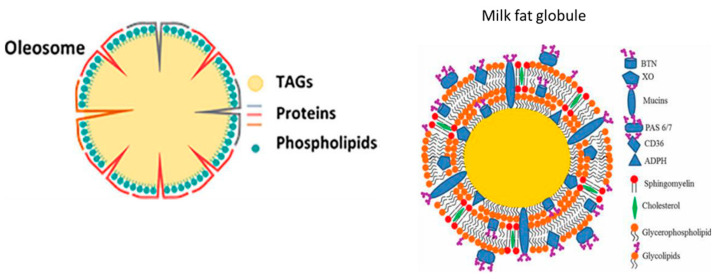
Tentative structure of oleosomes (plant-based beverages) and milk fat globules. From [12] with modifications.

In general, during the production of plant-based beverages, water extraction results in the formation of oil bodies, suspended solids (fibers), and plant proteins, creating a colloidal suspension with solids dispersed in a solution. To achieve uniformly sized particles in the solution, a homogenization process is carried out. Additionally, additives like vegetable oils (canola and sunflower oil) and vitamins could be incorporated into the beverage to enhance its emulsion stability, mouthfeel, texture, flavor, and overall nutritional value [10,11]. In plants, nuts and seeds have high lipid content, which can be extracted into oil bodies known as oleosomes during beverage production (Figure 1) [12]. Like bovine milk fat globules, oleosomes are typically between 0.2 to 2.0 μm in diameter, being spherical and containing a triglyceride core surrounded with a single layer of phospholipids (about 0.09 nm in thickness) in the membrane with integral proteins. Although oleosomes and milk fat globules are similar, they also differ in their membrane structure, which is multiple layers of phospholipids in milk fat globules, while in oleosomes, the phospholipids form a monolayer. However, they have the possibility of encapsulating hydrophilic and lipophilic compounds, so they are an interesting food delivery system for encapsulated bioactives. In this context, Brazil nut beverage was proposed as a potentially adequate and efficient plant food emulsion for OPD betalain and phenolic compounds’ encapsulation.

On the other hand, green plant extracts rich in bioactives could be considered food antioxidant ingredients to be added in order to improve the Brazil nut beverage’s health potential. One of these promising green extracts is obtained from cactus fruits such as *Opuntia stricta* var. *dillenii* fruits, which are a fruit native to the Canary Islands (Spain). The ODP green extract obtained from fruit pulp contains important bioactive compounds such as betalains (betacyanins and betaxanthins) and phenolic compounds with interesting antioxidant activities [12]. Betalains are nitrogen-based pigments responsible for the characteristic red color of the *Opuntia stricta* var. *dillenii* fruit pulp (ODP) due to the predominance of betacyanins. These compounds are very important since past studies have shown that they have antioxidant activity, and the presence of phenolic compounds in an OPD, such as phenolic acids (mainly piscidic acid) and flavonoids (mainly isorhamnetins and quercitin glycosides) have been demonstrated to help in weight loss processes by improving insulin resistance [13]. Phenolic compounds also perform an important anti-inflammatory activity. This means that prickly pear fruits (*Opuntia ficus-indica* and *Opuntia stricta* var. *dillenii*) have an important antioxidant and anti-inflammatory effect and have beneficial properties for obesity and diabetes treatments [14]. Recently, Gómez-López et al. [14] reported that the extracts from *Opuntia stricta* var. *dillenii* fruit tissues were effective in reducing triglyceride accumulation in murine mature adipocytes and reduced the activity of the ACC enzyme, assessed according to the ACC-Phospho/total ACC ratio. In addition, OPD pulp extract also reduced the expression of *fas*; both are involved in de novo lipogenesis, showing that a reduction in fatty acid uptake from the blood stream can also contribute to its triglyceride-lowering effect [14].

The aim of the present research was the preparation of healthy Brazil nut beverages enriched with green pulp extracts of *Opuntia stricta* var. *dillenii,* carrying out the physicochemical characterization of the enriched beverages as a food emulsion in which the bioactives from the green OPD extract were encapsulated. Also, a study of the stability and the changes in the individual encapsulated bioactive compounds content and antioxidant activity during the cold storage of the enriched BN beverages was also conducted to establish the best processing conditions to obtain a new plant-based beverage from Brazil nut and *Opuntia stricta* var. *dillenii* var. *dillenii* green extract with improved health potential due to the increase in the content of antioxidant compounds.

## 2. Materials and Methods

### 2.1. Plant Material

Brazil nuts, dry seed and shelled (without woody tegument), were purchased from a local market in Madrid (Spain) in April 2023. The vacuum-packaged Brazil nuts were stored in a refrigerated room at 4 °C. *Opuntia stricta* var. *dilleni* fruits were collected in Tinajo (Lanzarote, Canary Islands, Spain; 29° 3′ N, 13° 4′ W; 209 m over sea level). Fruits were selected based on their size, peel coloration, and ripeness. The damaged ones were discarded. The analysis of the physicochemical characteristics of fresh fruits was conducted; data are shown in Table S1 (Supplementary Materials). After washing, the selected fruits were manually peeled, and the pulp tissue (ODP) was stabilized via freezing with liquid nitrogen, freeze-dried, removed of all seeds, and stored at 24 °C until their use to obtain the ODP green extracts.

### 2.2. Chemicals

Ultra-pure water was obtained from a Milipak^®^ Express 40 system (Merk-Milipore, Darmstadt, Germany). Ethanol (99.97%) and the reagents formic acid and sodium carbonate were purchased from VWR International (Barcelona, Spain). The reagents potassium phosphate (KH_2_PO_4_), sodium phosphate (NaH_2_PO_4_), Trolox (6-hydroxy-2,5,7,8-tetramethylchroman-2- carboxylic acid), fluorescein, Folin–Ciocalteu, 2,20- azobis (2-methylpropionamidine) dihydrochloride (AAPH), trichloroacetic acid (TCA), and gallic acid were purchased from Sigma-Aldrich. Soy lecithin was purchased from VWR chemicals, and guar gum was purchased from MCS (Mexico City, Mexico). Betanin was extracted from commercial extracts of freeze-dried beets and purified using a Sephadex LH-20 resin purchased from Sigma-Aldrich (St. Louis, MO, USA). Phyllocactin was isolated from cactus berry fruits (*Myrtillocactus geometrizans*) using semipreparative, high-performance liquid chromatography (HPLC), as described by Gómez-López et al. (2021) [13]. Piscidic acid was purified from prickly pear peel extracts with semipreparative high-performance liquid chromatography (HPLC) using the protocol also described previously [13]. Isorhamnetin glycoside standards were supplied from the laboratory of Dr. Serna-Saldívar at the FEMSA Biotechnology center (School of Engineering and Sciences, Instituto Tecnológico de Monterrey, Monterrey, Mexico). Eucomic acid and its derivatives were quantified using the tyrosol standard [15]. Other phenolic compounds such as gallic acid, ferulic acid, protocatechuic acid, p-hydroxybenzoic acid, quinic acid, ellagic acid, p-coumaric acid, quercetin, catechin, epicatechin, vanillic acid, myricetin, rutin, and kaempferol were purchased from Sigma-Aldrich (St. Louis, MO, USA).

### 2.3. Extraction of Betalains and Phenolic Compounds in Pulp of Opuntia stricta var. dillenii

Extracts rich in betalains and phenolic compounds were obtained from freeze-dried *Opuntia stricta* var. *dillenii* (ODP) following the method of Gómez-López et al. (2021) [13]. Briefly, freeze-dried pulp was mixed with 5 mL of ethanol (1:1, *v*/*v*) and then centrifuged at 9000 rpm for 10 min to obtain the supernatant. This process was repeated three more times. The last extraction was carried out with 3 mL of pure ethanol. The final combined supernatants were vacuum-dried. The aqueous extract obtained was filtered with a 0.45 µm nylon filter (E0032, Analysis Vinicos, Ciudad Real, Spain). The *Opuntia dillenii* pulp extracts (ODP) obtained were freeze-dried and stored at −24 °C until HPLC analysis and their use to elaborate enriched Brazilian nut beverages (BNBs).

### 2.4. Elaboration of Brazil Nut Beverage with ODP Extract

The production of Brazil nut beverage was obtained following the method reported by Vazquez-Rojas et al. [8]. Briefly, the Brazil nuts were ground with a mortar or blender to reduce their size (particle size <5 mm). Then, they were homogenized with water at 75 °C at a ratio of 7:1 (water:Brazil nut, *v*/*w*) for 5 min at 750 rpm (OMNI Macro-ES Programmable Homogenizer, OMNI International, Kennesaw, GA, USA) until reaching a homogeneous consistency. Then, the solution was filtered with a stainless mesh (<2 mm) to obtain the aqueous extract. After it was cooled at 5 °C for 1 h in an ice bath, the extract was kept for 15 h in a refrigerator at 5 °C. After, the cream was removed (the upper part), and then, carefully (without stirring the sediment), the aqueous fraction was transferred to a new container, avoiding desedimentation. The aqueous phase (supernatant) involved the standardized (partially defatted) BNB used for the assay.

**Figure 2:**
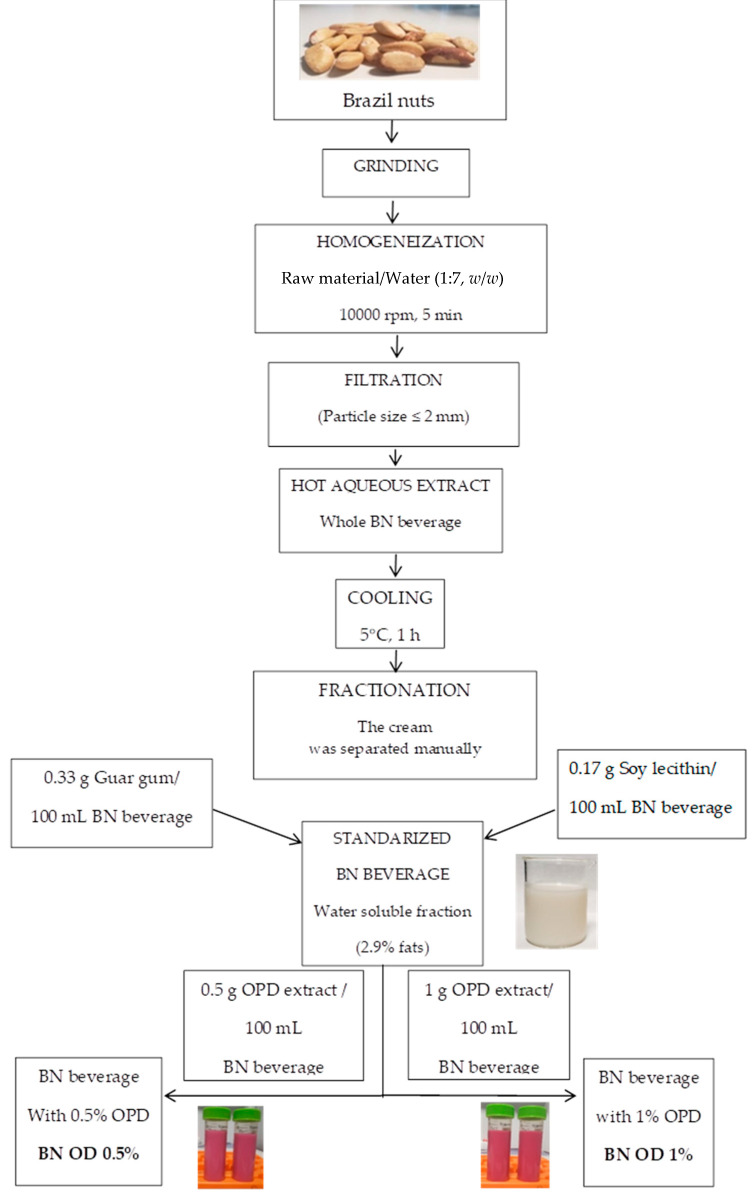
Flow diagram of the obtention of Brazilian nut beverage with *Opuntia stricta* var. *dillenii* pulp extract (ODP).

To stabilize the beverage and avoid phase separation, guar gum (0.33 g of guar gum/100 mL of beverage) and soy lecithin (0.17 g of soy lecithin/100 mL of beverage) were added as stabilizers, following the recommendations on other plant-based beverages [15]. After obtaining the stabilized Brazil nut beverage, the *Opuntia dillenii* pulp extracts (ODPs) were added, as indicated in Figure 2. Two amounts of *Opuntia dillenii* extract were used to enrich the Brazil nut beverage, 0.5 g or 1.0 g of ODP extract to 100 mL of stabilized Brazil nut beverage.

### 2.5. Cold Storage of ODP-Enriched Brazil Nut Beverages

After the preparation of the Brazil nut beverages with ODP extracts and without (the control sample), all of them were stored for 24 days at 5 °C. Samples were taken at days 1, 3, 5, 8, 12, and 24 to analyze the emulsion stability and determine the evolution of the bioactive compounds and antioxidant activity.

### 2.6. Extraction of Betalains and Phenolic Compounds from Brazil Nut Beverages with ODP Extract

For the extraction of betalains and phenolics compounds from BN+ODP beverages, a solution of trichloroacetic acid (4% TCA in water) was used, according to the method proposed by Naderi et al. [16]. Briefly, 4 mL of the beverage was poured into a 15 mL tube. Then, 4 mL of trichloroacetic acid solution (4% TCA in water) was added in a 1:1 ratio (beverage:TCA, *v*/*v*), Next, the mixture was vortexed for 3 min and then centrifuged for 10 min at 6000 rpm at 25 °C. The supernatant was filtered with a 0.45 µm filter and then injected into the HPLC equipment to determine the content in the main individual bioactives (betalains and phenolic compounds) and to calculate their encapsulation efficiencies. The extraction procedure was carried out in triplicate for all samples (Brazil nut beverage (BNB), Brazil nut beverage with 0.5% ODP extract (BN ED 0.5%), and Brazil nut beverage with 1%. ODP extract (BN ED 1%)).

### 2.7. Methods of Analysis

#### 2.7.1. Characterization of Betalains and Phenolic Compounds of ODP Extract

For the simultaneous characterization of individual betalains and phenolic compounds, the extracts of *Opuntia stricta* var. *dillenii* (ODP) pulp were dissolved in ultra-pure water and filtered through 0.45 µm nylon filters using the method reported by Gomez-Lopez et al. [13]. Briefly, the 1200 Series Agilent HPLC System (Agilent Technologies, Barcelona, Spain) with a C18 reverse column Zorbax SB-C18, 250 × 4.6 nm, i.d., S-5 µm (Agilent Technologies, Santa Clara, CA, USA) at 25 °C was employed. Ultra-pure water with 1% formic acid (*v*/*v*) (Phase A) and methanol (99.8% LC-MS) with 1% formic acid (*v*/*v*) (Phase B) was used in a gradient for 70 min in order to obtain the optimal separation of bioactive compounds. A flow rate of 0.8 mL/min and an injection volume of 20 µL were used. The UV–visible photodiode array detector was set at four wavelengths: 280 nm for phenolic acids, 370 nm for flavonoids, 480 nm for betaxanthins, and 535 nm for betacyanins. The chemical characterization of each compound was determined from the data obtained with UV-vis and MS-QT detectors and compared with their real standards. Quantification was performed from calibration curves obtained with these real standards via UV-vis detection. Gómez-López et al. [13] reported the complete description of UV-vis and mass spectroscopy characteristics of all individual betalains and phenolic compounds found in ODP green extracts.

#### 2.7.2. Physicochemical Analysis of *Opuntia stricta* var. *dillenii* Fruits, Brazil Nuts, and the Beverage

The composition (ash, moisture, and dry matter) of the Brazil nuts, the *Opuntia stricta* var. *dillenii* fruits, and the Brazil nut beverage was analyzed using the standardized AOAC method [17]. For the determination of proteins, the chemical analysis method of nitrogen was used with 6.25 as a conversion factor. For the determination of lipids, the Folch method was used. Regarding the physicochemical properties, the pH was directly measured for the beverage with a digital potentiometer (Metrohm 827 pH Meter, Metrohm, Herisau, Switzerland). The titratable acidity (g of citric acid/100 mL of beverage) was analyzed via the neutralization of the beverage, measuring the volume of 0.1 N sodium hydroxide expenditure until reaching a pH of 8.1. Soluble solids (°Brix) were determined directly from the beverage using a digital refractometer (PR-32, ATAGO™, Tokyo, Japan). All information about the physicochemical characteristics of Brazil nuts, *Opuntia stricta* var. *dillenii* fruits, and the Brazil nut beverage are shown in Table S1 (Supplementary Materials). The pH, soluble solids (°Brix), and acidity were also measured for the Brazil nut beverages with added pulp extracts of *Opuntia stricta* var. *dillenii* (ODP).

#### 2.7.3. Color Measurement of *Opuntia stricta* var. *dillenii* Fruit Pulp and Brazil Nut Beverages with ODP Extracts

The color measurement was performed with a colorimeter (SPECORD 210 PLUS, Analytik Jena, Jena, Germany) using the CIELab color space method, for which L* was the luminosity, a* was the (red–green) tonality color, and b* was the (blue–yellow) tonality color with an illuminant of D65 and a standard viewing angle of 10 with a spectral scan from 380 to 780 nn. The total color differences ∆E (Equation (1)) were calculated during the cold storage of the BN beverages with ODP extract while using the BNB (Brazil nut beverage without ODP extract (control)) as the reference.

∆E = (∆L*^2^ + ∆a*^2^ + ∆b*^2^)^1/2^ (1)

#### 2.7.4. Optical Microscopy of Brazil Nut Beverages

In order to study the microstructure of Brazil nut beverages enriched with *Opuntia stricta* var. *dillenii* pulp extracts (ODP), an optical microscope (Leica DM2500, Wetzlar, Germany) was used. This study was carried out during cold storage at 5 °C for the Brazil nut beverages. The amplitude to take the images was 40×, and they were captured using the Leica DFC295 camera (Leica Microsystems, Wetzlar, Germany). The images were processed using the device’s software (LAS version 4.13.0, Leica).

#### 2.7.5. Particle Size and Zeta Potential Measurements

The Zetasizer Pro equipment (Malvern Instruments Ltd., Worcestershire, UK) was used, a dynamic light scattering system (DLS) for the measurement of the particle size of Brazil nut beverages (as they are emulsions) and an electrophoretic light scattering system (ELS) for the measurement of their Z potential. To measure both parameters, 100 µL of each sample (Brazil nut beverages with and without ODP extract) were taken and diluted in 10 mL of MilliQ water. Then, the samples were homogenized and transferred to cuvettes inside an equipment chamber. The measurements were made under time and temperature parameters of 120 s at 25 °C with a dispersion angle of 174 and a refractive index of 1.45. The measurement process was carried out in triplicate for all BN samples and during their cold storage.

#### 2.7.6. Analysis of Total Phenolic Content

The total phenolic content was determined via the spectrophotometric method reported by Gómez-López et al. [13]. Briefly, 75 μL of MiliiQ water was added to 75 μL of the sample (extracts of Brazil nut beverage with ODP) at a ratio of 1:1 (water:beverage, *v*/*v*). Then, 750 μL of Follin reagent, previously diluted in MiliiQ water, was added (dilution: 1:10, Follin reagent:water *v*/*v*). Then, the mixture was homogenized in a vortex and allowed to stand for 5 min. Next, 600 µL of Na_2_CO_3_ 7.5% (*w*/*v*) was added. The control was evaluated in the same way but using 150 µL of miliiQ water instead of the sample. This procedure was performed in triplicate, and finally, the samples were incubated for 30 min in darkness, and spectrophotometric measurements were carried out using equipment (Varioskan Flash, Thermo Fisher Scientific, Waltham, MA, USA) at 760 nn. To carry out the calibration curve, gallic acid was used as a reference standard at a concentration of 50 to 1000 µg/mL, and the results were expressed in mg equivalents of gallic acid per 100 mL of beverage (mg GAE/100 mL beverage). Additionally, the same analysis protocol was carried out to determine the total phenolic content directly in the beverage samples, without a previous extraction, in order to study the possible differences among the measurements performed when a previous extraction was carried out. The results for the total phenolic content obtained from Brazil nut beverages without previous extraction are shown in Table S4 (Supplementary Materials).

#### 2.7.7. Antioxidant Capacity ORAC Assay

The oxygen radical antioxidant capacity (ORAC) method was used for the determination of the antioxidant activity, taking as a reference the method carried out by Gómez-Maqueo et al. [18] with some modifications. Briefly, the samples were dissolved with a phosphate-buffered saline solution (PBS) of 75 mM at a pH of 7.4. The preparation of the Trolox curve was done in concentrations of 100 to 450 µL/mL in a microwell plate; 20 µL of the sample extract was placed in each microwell. Then, 120 µL of fluorescein was added to all the wells of the microplate, and the microplate was incubated at 37 °C for 10 min. A solution of 153 mM of 2,20-Azobis (2-ethylpropionamidine) dihydrochloride (AAPH) was prepared, and after incubation, 60 µL of AAPH was added to the samples. The equipment employed for spectrophotometric analysis was a Varioskan Flash (Thermo Electron Corporation^®^, Waltham, MA, USA). Readings were recorded each minute for 95 min. Data analysis was performed by finding the area under the curve (AUC) minus the blank. The results were expressed as µmol TE/g BN of beverage or µmol TE/g of ODP extract. Additionally, the same protocol was employed to analyze the antioxidant capacity directly in the BN beverages without previous extraction, as reported in Section 2.7.6, for total phenolic analysis (Section 2).

#### 2.7.8. Statistical Analysis

The processing of the data obtained in the present work was conducted using the Statgraphics 19 statistical program (Statgraphics Technologies, Inc., The Plains, VA, USA). For the evaluation of the variables studied in the different groups, the analysis of variance (ANOVA) was used with a significant difference of *p* < 0.05, and for the evaluation, the Tukey test was used to determine the differences between means. For all the assays carried out in the present research, the samples were processed and analyzed in triplicate.

## 3. Results and Discussion

### 3.1. Characterization of Bioactives Compounds from Opuntia stricta var. dillenii Fruit Pulp Extract (ODP)

The characterization of the betalains and phenolic compounds present in the pulp extracts of *Opuntia stricta* var. *dillenii* (ODP) was carried via the HPLC. The identification of these bioactives was achieved while taking into account their retention times, UV-vis data, and mass spectrum data, as reported previously by Gómez-López et al. [13].

Among the main bioactive compounds, the betalain betanin (peak 7) was found to be the most abundant betacyanin in the ODP extracts, followed by isobetanin (peak 8) and neobetanin (peak 14), as well as two isomers of neobetanin (neobetanin isomer II and neobetanin isomer III); see Table 1 and Figure S5 (Supplementary Materials). Other betalains such as 2′-O-Apiosyl-4-O-filocactin (peak 12) and 5″-O-E-Sinapoyl-2′- apyosyl-filocactin (peak 13) could also be found in the OPD extract in low quantities; see Figure S3 (Supplementary Materials). Regarding the main phenolic compounds, piscidic acid was found (peak 6) to be the most abundant. In addition, other minor organic acids were found, such as ascorbic acid (peak 1), citric acid (peak 2), quinic acid (peak 3), and euchomic acid (peak 9); see Table 1 and Figure S2 (Supplementary Materials).

Finally, among the flavonoids, Isorhamnetin glucoxyl-rhamnosyl-rhamnoside (IG1) (peak 19) and Isorhamnetin glucoxyl-rhamnosyl-pentoside (IG2) (peak 20) were also found in the OPD extract; see Table 1 and Figure S4 (Supplementary Materials). The complete composition of the betalains and phenolic compounds in the pulp extract of *Opuntia stricta* var. *dillenii* fruits was reported previously by Gómez-López et al. [13].

### 3.2. Physicochemical Characteristics of Opuntia stricta var. dillenii Fruits, Brazil Nuts and Beverage

The composition of the Brazil nut and the Brazil nut beverage is shown in Table S1 (Supplementary Materials). The composition of the Brazil nuts showed high total proteins (17.3%), total carbohydrates (10.9%), and total lipids (66.1%) content, like most other nuts [19,20,21]. However, the Brazil nut lipid content is among the highest compared to other nuts, such as cashews, peanuts, pistachios, almonds, walnuts, and hazelnuts [8]. Most of the lipids of Brazil nut are unsaturated fatty acids; in addition to this, it has other phytochemical compounds, such as selenoammonium acid, dietary fiber, minerals, phenolic compounds, tocopherols, and phytosterols, which provide a potential health benefit related to the potential prevention of cardiovascular diseases, the prevention of cancer, and an improvement in cognitive functions [8].

With respect to proteins, other nuts, such as almonds and peanuts, have a higher content than Brazil nuts [19]. The composition of the standardized Brazil nut beverage obtained in the present study is shown in Table S1 (Supplementary Materials). The fat content of the standardized BN beverage was 2.9%, which is a similar value to that found in other commercial plant-based beverages [20,21]. To obtain this total fat content in the standardized BN beverage, a controlled defatting or skimming process was carried out; see Figure 2.

Significant changes in their physicochemical characteristics were observed in the Brazil nut beverages after the addition of the ODP extract (day 0); see Table 2. In general, the pH decreased in the BN+ODP beverages, and the acidity and soluble solids increased: the Brazil nut with 1% ODP extract BN beverage showed the lowest pH (4.44 ± 0.1) and the highest acidity (0.15 ± 0.00 g citric acid/100 mL beverage) and soluble solids (°Brix) (3.75 ± 0.21); see Table 2. This fact was expected since the ODP extract contains 36.7 ± 5.7 g/100 g of *O. dillenii* pulp of total carbohydrates and an acidity of 1.58 ± 0.1 g citric acid/100 g of *O. dilleni* pulp (Table S1, Supplementary Materials), which logically contributed to the modification of these parameters in the BN beverages with ODP extracts. Table 2 also shows the data for all the physicochemical characteristics of Brazil nut beverages (BNBs) and Brazil nut beverages with 0.5% (BN ED 0.5%) and 1% (BN ED 1%) ODP extract during their storage at 5 °C for 24 days.

These results are in accordance with some studies previously reported by Dimitrellou et al. [22], in which, in a beverage made from wheat that was enriched with fruit juices, a pattern of acidification was observed, and at the same time, a decrease in pH was observed compared to wheat beverages without added juices. At the same time, previous studies carried out on kefir-based beverages in which fruit juice was added also showed a higher acidity and pH after the addition of the juice [23] due to the low pH of the fruits causing the reduction in the pH in the medium in which they were added to decrease, and at the same time, causing an increase in acidity. In the present work, this trend was observed throughout the 24 days of storage at 5 °C for BN beverages with ODP extract; see Table 2.

The color parameters of the BN beverages without and with ODP extracts (at two concentrations) during cold storage at 5 °C are shown in Table 2. When taking into account the color parameters L*, a*, and b*, the Brazil nut beverage without ODP extract showed the highest values of luminosity (L*), and this luminosity remained almost unchanged throughout the 24 days of storage at 5 °C, and the BN beverage was just prepared (day 0) when this beverage exhibited the highest luminosity value, L* = 27.55 ± 0.86; see Table 2. Similar trends were observed in previous reported studies carried out on soy yogurts that were enriched with extracts of red beet, opuntia, and red radish, which reported that soy yogurt without extracts added showed the highest luminosity value [24].

The Brazil nut beverage with 0.5% ODP extract showed the highest values of the a* color parameter (degree of redness and greenness), 11.88 ± 2.69. In contrast the b* color parameter (degree of yellowness and blueness) was higher in the Brazil nut beverage with 1% ODP extract, at 3.06 ± 0.25, also at day 0 (Table 2). This fact could be due to a higher a* value on the red–green axis not necessarily being perceived as a redder color in the food since the hue is not only defined by the a* or b* values. These data agreed with the studies carried out by Prieto-Santiago et al. [25] in which, in different beetroot samples, the samples with highest content of betacyanins showed lower values for a* and higher values for b*. These two trends in parameters a* and b* for BN beverages with OPD extracts did not show any significant changes during cold storage at 5 °C for 24 days; see Table 2.

In general, the color differences (∆E) among the BN beverages with ODP extracts and the BN beverage without an ODP extract (the control) were similar in both drinks (BN+0.5%ED and BN+1%ED); the BN beverage with 0.5% ODP extract (BN+0.5%ED) was the beverage that showed a slightly greater color difference precisely due to the lesser amount of ODP added. With respect to the storage, the BN beverages did not show any significant changes throughout the study conducted at 5 °C.

### 3.3. Microstructure, Visual Appearance, and Stability of BN Beverages

Conventionally high-shear mixers and homogenizers are used with a loaded number of chemical stabilizers and emulsifiers to stabilize plant beverages. In the present study, guar gum (0.33 g of guar gum/100 mL of beverage) and soy lecithin (0.17 g of soy lecithin/100 mL of beverage) were added as stabilizers, following the recommendations of a reported study about obtaining plant-based beverages [2]. The loading of these emulsifiers in the standardized BN beverage was conducted using a high-speed homogenizer, producing a very stable food emulsion.

Figure 2 shows the images obtained from the optical microscope and the appearance of both BN beverages (with 0.5% and 1.0% ODP extract added). In these images, purple–red fat drops (oleosomes) of different sizes at the edges can be seen, and they are much darker in the BN with 1% ODP extract. These fat globules (oleosomes) showed red zones inside themselves due to the presence of the betalains from the encapsulated ODP extract. Dark-colored globule conglomerates were also observed in BN beverages with ODP extracts during cold storage; see Figure 2. Some visual differences were observed in the color of BN beverages; the BN beverage with 1% ODP extract added was the sample that showed a stronger red color.

The BN beverages remained stable throughout the 24 days of storage at 5 °C according to visual appreciation. No type of phenomenon, such as gravitational separation (cream and/or sedimentation), aggregation (flocculation and/or coalescence), or phase separation (greasing), was observed. This observed stabilization of the BN beverages was due to the addition of stabilizers (guar gum and soy lecithin) in their elaboration process. In a previous study carried out by Vazques-Rojas et al. [8], a Brazil nut beverage elaborated without the addition of stabilizers showed an aggregation phenomenon and phase separation during cold storage.

### 3.4. Particle Size and Zeta Potential of BN Beverages

**Figure 3:**
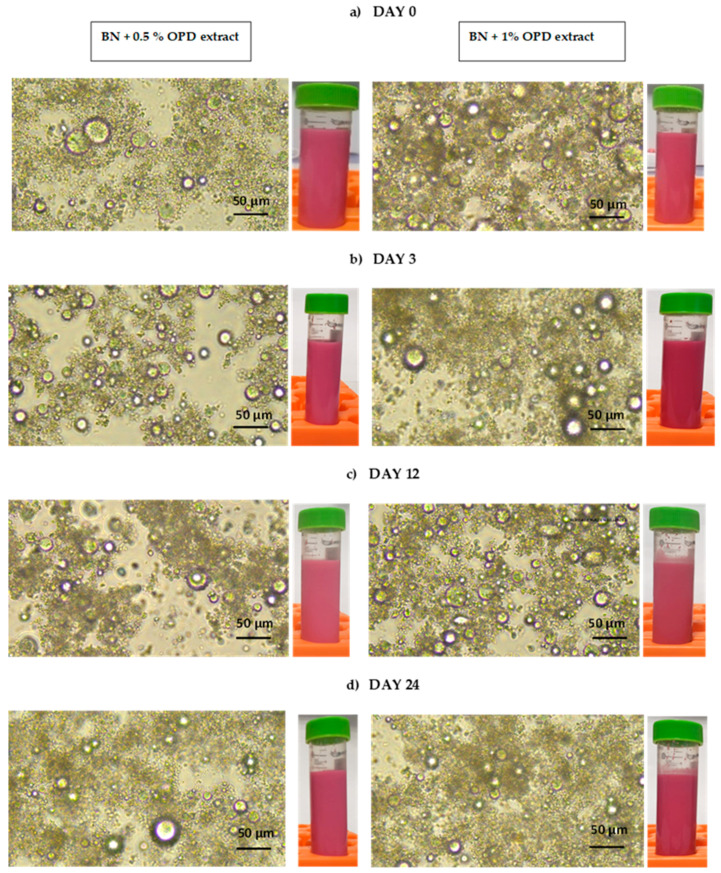
Visual appreciation and optical microscopy images of the Brazil nut beverage with *Opuntia stricta* var. *dillenii* pulp extract during cold storage at 5 °C for 24 days. On the right, the Brazil nut beverage with 1% ODP extract, and on the left, the Brazil nut beverage with 0.5% ODP extract at (**a**) day 0, (**b**) day 3, (**c**) day 12, and (**d**) day 24 of cold storage.

**Figure 4:**
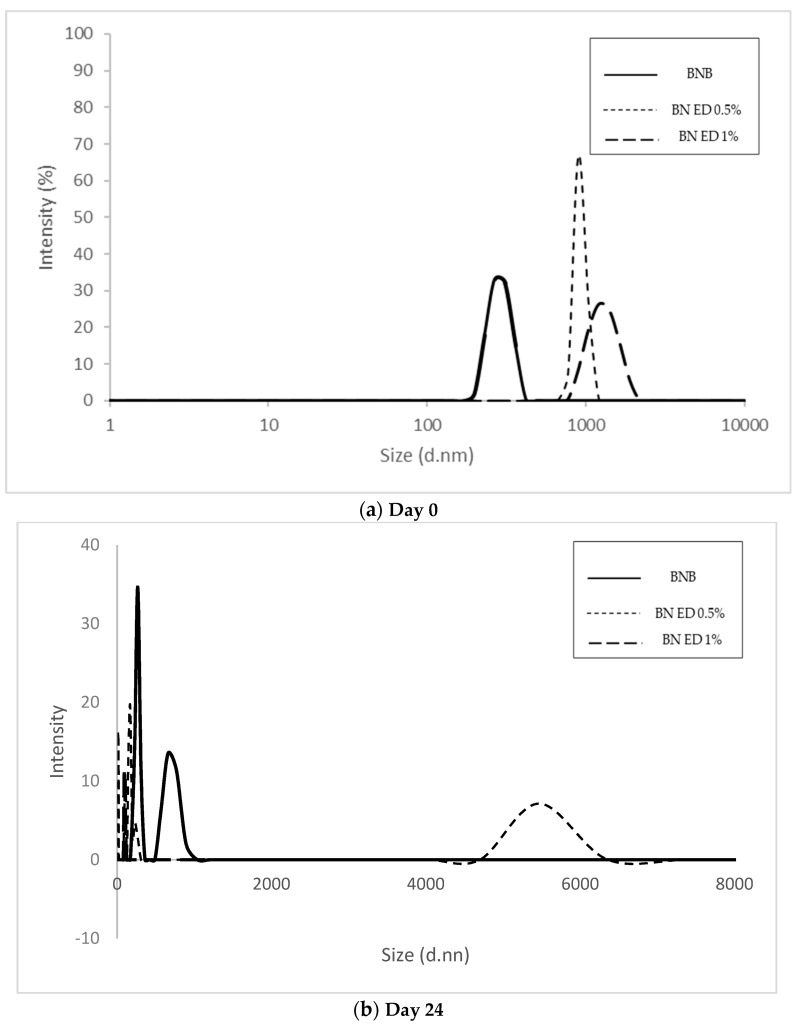
Particle size distribution of Brazil nut beverage (BNB) with *Opuntia stricta* var. *dillenii* pulp extract during cold storage preserved at 5 °C for 24 days. (**a**) day 0 and (**b**) day 24 of cold storage.

Particle size is a very important factor to take into account concerning the stability of an emulsion. This is because maintaining a small particle size in plant-based beverages represents one of the most effective ways to avoid gravitational separation and, therefore, guarantee a longer shelf life. In general, it is recommended that the particle size diameter in plant-based beverages should not exceed 300 nn (D < 300 nn) [26]. Table 3 shows the particle size and zeta potential data in the Brazil nut beverages obtained in the present study (the BN beverage without ODP extract, the Brazil nut beverage with 0.5% ODP extract, and the Brazil nut beverage with 1% ODP extract). Among these BN beverages, the Brazil nut beverage without ODP extracts (the control) was characterized by the lowest particle size values at 861 ± 80 nm on day 0 (for the just-prepared BN beverage). This particle size value continuously increased up 8.5 times (7554 ± 263 nm) at 24 days of cold storage at 5 °C, Figure 3 and Figure 4.

Regarding Brazil nut beverages with ODP extracts, the BN beverage with 1% ODP extract showed the highest particle size values after it was just elaborated and during cold storage; see Table 3. Figure 3 and Figure 4 shows the particle size distribution graphics of BN beverages just elaborated (day 0) and at the end of storage at 5 °C (24 days).

In the case of the Brazil nut beverage without ODP extract (the control), the particle size values were lower than those reported by Vasquez-Rojas et al. [8] for BN beverages ellaborated without emulsifiers but treated with high hydrostatic pressurization to preserve them. This fact could be related precisely to the use of gum guar and soy lecithin as stabilizers in the present study, in which the particle size values were higher for both BN beverages without ODP extract (control) and beverages with ODP extract.

The zeta potential is another factor to consider in the stability of an emulsion. It measures electrical interactions in food systems and is defined as the potential between the liquid layer immediately next to the surface (strongly attached to the particle) and the continuous phase. In the case of emulsions, to guarantee its stability, the zeta potential is considered an absolute value less than or greater than 25 mV [27,28].

As shown in Table 3, the BN beverage without the extract showed stable values of Z potential until day 12 of storage. With respect to the BN beverages with ODP extracts, only the BN beverage with 0.5% ODP at day 0 (the day of preparation) showed Z potential values within the acceptability threshold (less than or greater than 25 mV) [28]. Both of the BN beverages with OPD extract added showed a progressive decrease in Z potential values during cold storage at 5 °C, indicating the certain instability of the emulsions that was not visually observable; see Figure 2.

### 3.5. Characterization of Main Individual Betalains and Phenolic Compounds in Brazil Nut Beverage with Added ODP Extract

The extracts of the BN beverages contained the most abundant original phenolic compounds that are typical of Brazil nuts, which were detected at λ_max_ 280 nm. It was also possible to identify, together with the phenolics, some organic acids, such as succinic acid (peak 4), gallic acid (peak 5), and p- coumaric acid (peak 16); see Table 4 and Figure S2 (Supplementary Materials). In addition, flavonoids such as catechin and its derivative belonging to the flavan-3-ol subgroup were detected, and taxifolin (pico18), belonging to the flavanonol subgroup, was also detected in BN beverages; see Table 4 and Figure S3 (Supplementary Materials). All of these compounds found in the BN beverages agreed with the phenolic compounds reported by some authors for this plant-based beverage [29]. Regarding the compounds from the encapsulated ODP extract, some phenolic acids such as piscidic acid (peak 6) could be identified together with some organic acids such as ascorbic acid (peak 1) and eucomic acid (peak 9); see Table 4, detected at λ_max_ 280 nm.

Among the flavonoids, compounds such as isorhamnetin glucoxyl- rhamnosyl-rhamnoside (IG1) (peak 19) and isorhamnetin glucoxyl-rhamnosyl-pentoside (IG2) (peak 20) could be identified in the analysis of BN+ODP beverages; see Figure S3 (Supplementary Materials). Betalains were identified and detected at λ_max_ 480 nm (betaxanthnis) and λ_max_ 535 nm (betacyanins). Betanin (peak 7) and isobetanin (peak 8) were the most abundant compounds, followed by neobetanin (peak 14), as well as isomers of neobetanin such as neobetanin isomer II (peak 15) and neobetanin isomer III (peak 17); see Figures S4 and S5 (Supplementary Materials) and Table 4.

All the identified bioactive compounds found in the BN beverages with ODP extract added are shown in Table 4 and Figures S2–S5 of the Supplementary Materials, which show the obtained typical HPLC chromatograms.

### 3.6. Content of the Main Individual Betalains and Phenolic Compounds in BN Beverages with ODP Extract

Table 5 shows the content of the main betalains and phenolic compounds in the Brazil nut beverages with ODP extract analyzed while carrying out the specific extraction of these bioactive compounds for HPLC analysis (see Section 2). Betacyanins were the betalains that were found with the highest content in the OPD pulp extracts, in which betanin (8188.12 µg/gr of extract), followed by isobetanin (5042.18 µg/gr of extract), was the most abundant compound. These findings are in agreement with several previous studies carried out by our research group on *Opuntia stricta* var. *dillenii* fruits [13]. These authors reported that the pulp was the fruit tissue with the highest content of these betacyanins. In the present study, neobetanin content was also found in the ODP extracts, but at a low level (4039.62 µg/gr of extract) compared to the betanin and isobetanin ones.

Regarding the phenolic compounds, piscidic acid (4262.18 µg/gr of extract) was the most abundant compound found in the ODP extracts, and with respect to the flavonoids, only small amounts of glucoxyl-rhamnosyl-rhamnoside (IG1) and isorhamnetin glucoxyl-rhamnosyl-pentoside (IG2) were quantified (17.46 µg/gr of extract and 27.59 µg/gr of extract, respectively); see Table 5. This fact was because the *Opuntia stricta* var. *dillenii* fruit flavonoids are mainly present in the fruit peel [13].

Previous studies reported the use of betalains in foods with different formulations, especially as a natural colorant with antioxidant and antimicrobial activities [30]. Regarding the use of betalains in beverages, Dabija et al. [31] reported the addition of beet powder in natural yogurt, evaluating its effect on the physicochemical, rheological, and sensory properties of the yogurt. Likewise, Dias et al. [32] studied the color stability of fermented soy yogurts with added beet, opuntia, and red radish extracts and evaluated the behavior of the pigments (betalains) during storage.

In general, in the present study, few losses were observed in the content of betalains and phenolic compounds during storage (Table 5), maintaining high recovery percentages in both the BN beverages with ODP extracts throughout the 24 days of storage at 5 °C. This occurred despite the fact that previous studies indicated that, in food products such as beet puree refrigerated at temperatures of less than 24 °C, the betanin content decreased by 21.5% after 7 days of storage [33]. At the same time, studies carried out on red beet juices and nectars reported progressive losses of 46.9% in betalain content after 60 days of storage at 5 °C [34,35].

### 3.7. Encapsulation Efficiency of Main Betalains and Phenolic Compounds of OPD Extract in BN Beverages

The individual encapsulation efficiency of each individual main betalain and phenolic compounds present in the BN beverages with ODP extracts are shown in Table S2 (Supplementary Materials). The encapsulation values are important because they indicate the health potential of the enriched BN beverage, showing the real amount of each bioactive compound from the OPD extract available in the BN beverage because not all amounts of each bioactive present in the added extract were equally solubilized during the beverage elaboration process. High encapsulation efficiencies were observed for all of the most bioactive compounds. The data for the EE% of the bioactives from the BN beverage with 1% ODP showed the highest values (betanin: 98.84%; piscidic acid: 93.67%; IG2: 95.40%); see Table S2 (Supplementary Materials). However, all values for encapsulation efficiency decreased during the cold storage of the BN beverages, probably due to the different stability of each bioactive. These results are in accordance with those reported by Güneser [34], who studied cow’s milk colored with beet extracts, finding that the recovery of betalains (betanin and isobetanin) was greater than 90% after extraction. In the present research, neobetanin was the betalain with the lowest encapsulation efficiency. This fact could be due to the low quantity of this bioactive in the ODP extracts, which made their extraction for analysis more difficult. Phenolic compounds also showed high encapsulation efficiency values in both BN beverages, with data such as 93.67% and 81.20% for piscidic acid in the BN beverages with 1% and 0.5% ODP pulp extract, respectively.

In the present study, many factors must be considered to explain observed slight losses in the content of betalains and phenolic compounds during the storage of the BN beverages. BN beverages act as emulsions with a high capacity to encapsulate the bioactive compounds of ODP extract. Also, another important factor is that the pH of the BN beverages was in the range of 4.7–5.8, a pH range at which betalains are very stable [35,36]. Additionally, previously published studies reported that the presence of certain compounds such as ascorbic acid and selenium in a BN beverage could have a beneficial influence on improving betalains’ stability at 5 °C [35]. And finally, a last factor to be considered is that, according to previous studies carried out on red beets, some preservatives such as pectin and guar gum could also improve the stability of betalain compounds [36].

### 3.8. Total Phenolic Content (TPC) of BN Beverages and BN Beverages with ODP Extract

The total content of phenolic compounds analyzed via the Folin–Ciocalteau method was also studied in order to compare the obtained data on BN beverages with added ODP extract to the reported data of previously published studies conducted on other plant-based beverages. The TPC in the Brazil nut beverage without OPD extract was 13.57 mg GAE/100 mL; this value was higher than those reported by Vasquez-Rojas et al. for this same BN beverage (7.1 mg of GAE/100 g) [8]. Brazil nut beverages showed higher total phenolic content than other commercial beverages such as rice, peanut, and coconut, among others, which ranged between 0.02 and 1.24 mg of GAE/100 mL [37].

**Figure 5:**
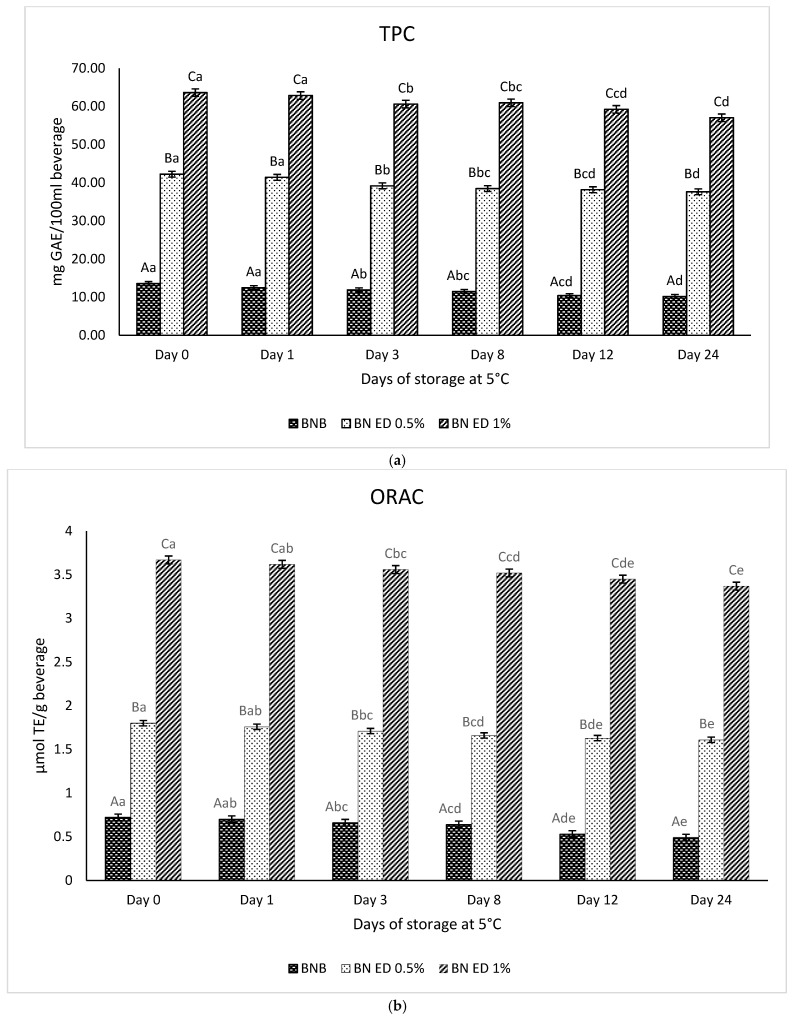
(**a**) Total phenolic content (TPC) and (**b**) oxygen radical absorbance capacity (ORAC) of Brazil nut beverages (BN) and BN beverages with 0.5% (BN ED 0.5%) and 1% (BN ED 1%) of *Opuntia stricta dillenii* pulp extract added during storage at 5 °C for 24 days analyzed after the extraction method of Naderi et al. (2010) [24] using trichloroacetic acid (TCA). Different uppercase letters indicate statistically significant differences (*p* < 0.05) among samples at the same storage time. Different lowercase letters indicate significant differences (*p* < 0.5) in storage times for the same sample.

Figure 5a shows the evolution in the content of total phenols (TPC) in the BN beverages with and without added OPD pulp extract. Regarding the Brazil nut beverages with OPD pulp extracts, significant differences could be observed with respect to the BN beverage without ODP extract; the Brazil nut beverage with 1% added OPD pulp extracts was the sample with the higher content of 63.61 mg of GAE/100 mL at day 0 (just after BN elaboration). Logically, the addition of 1% ODP extract contributed to the TPC value of the BN beverage because the total phenol content of the OPD extract was very high due to its composition in phenolic acids and flavonoids (Table 5). Previous studies carried out on prickly pear fruits from *Opuntia ficus-indica* and *Opuntia stricta* var. *dillenii* reported a high content of phenols in their fruit tissues, mainly in the fruit peel [13,38,39].

Significant differences were observed when comparing the measurements of total phenols in the BN beverage with 1% ODP, according to the sum of the individual phenolic content analyzed via HPLC (16.48 mg/100 mL of BN beverage) and the one obtained via the Folin–Ciocalteau method (63.61 mg/100 mL of BN beverage). This fact agrees with previously reported studies in which the trend indicated that the total phenol content was higher according to the Folin–Ciocalteu method than the HPLC method [40,41], indicating that the spectrophotometric method is not totally specific for phenolic compounds, and it is usually used only for the purpose of comparing extracts via a rapid and cheap analysis. To achieve an accurate measurement of the amount of total phenolic compounds, the chromatographic method must be used because it is more sensitive and specific than the spectrophotometric method. Additionally, in plant extracts, other interfering co-extracted compounds, such as sugars and ascorbic acid, would contribute to the total phenolic content analyzed via the Folin–Ciocalteau method [42]. Likewise, in the present study, total phenol content was also determined directly in Brazil nut beverages without previous extraction, and the data are shown in Table S4 (Supplementary Materials).

### 3.9. Antioxidant Capacity of BN Beverage and BN Beverages with ODP Extract

The oxygen radical scavenging capacity (ORAC) assay was used to determine the antioxidant capacity of the BN beverages. Figure 5b shows the evolution of the antioxidant capacity in BN beverages during 24 days of storage at 5 °C. The antioxidant capacity of the Brazil beverage without ODP extracts was 0.72 µmol of TE/g of BN beverage, which was the lowest among all of the elaborated BN beverages. Low antioxidant capacity values were found in previously reported studies on Brazil nut beverages with 0.47 µmol of TE/g [8].

BN beverages with ODP extracts showed very high values for antioxidant capacity; the BN beverage with 1% ODP extract added was the sample with higher data (3.67 µmol of TE/g of beverage). Logically, the addition of ODP extract considerably improves the antioxidant capacity of the BN beverages by more than 5-times; the BN beverage without OPD extract showed a considerable antioxidant capacity due to its bioactive composition, mainly concerning phenolic compounds and selenium [8]. When OPD extract was added to this BN beverage, the antioxidant capacity of the formulated BN beverage was improved. Previous studies on the antioxidant capacity of prickly pear fruits indicated that the extracts obtained from different fruit tissues showed different values; the red–purple Opuntia and the *Opuntia stricta* var. *dillenii* varieties were those that exhibited higher antioxidant capacity data [18,38,42].

A reduction in the antioxidant capacity values was observed during the 24 days of cold storage in all the BN beverages; see Figure 5b. This fact could be due to small decreases observed in the content of bioactive compounds such as betalains and phenolic compounds (Table 5), which are mainly responsible for the antioxidant capacity of a BN beverage. Previous studies reported the direct relation between betalains and phenolic compounds and the antioxidant capacity [3,42,43]. The data on the evolution of the antioxidant capacity of all the BN samples during the 24 days of storage at 5 °C are shown in Table S3 (Supplementary Materials). In the same way, the antioxidant capacity was determined directly as the total phenolic content without previous extraction; the results are shown in Table S4 (Supplementary Materials).

## 4. Conclusions

The present research study has shown that a standardized Brazil nut beverage with guar gum and soy lecithin as stabilizers is a very interesting beverage food emulsion to encapsulate *Opuntia stricta* var. *dillenii* green extract in order to obtain a healthy plant-based beverage with a high antioxidant capacity. BN beverages behave like emulsions in which their oleosomes have the ability to efficiently encapsulate the bioactive compounds (betalains and phenolic compounds) of OPD extracts. The elaborated BN beverages with OPD extracts were stable during cold storage at 5 °C for 24 days, showing little aggregation of their particles that could be observed via optical microscopy, but this fact did not affect the stability of the beverages. In general, good encapsulation efficiencies were obtained for the most abundant ODP bioactives encapsulated in the BN beverages during storage, mainly due to the intrinsic characteristics of the BN beverages like their composition, pH, and added stabilizers. All of this together promoted the stability of the ODP-added compounds and, at the same time, protected the high antioxidant capacity of the ODP extract, improving the health potential of the Brazil nut beverages. Additional research must be conducted to study the improvement in the bioaccessibility of the ODP and BNB bioactives present in the beverage in order to best define the nutritional and health potential of this plant-based beverage.

## Figures and Tables

**Table 1:** Characterization of the most abundant individual betalain and phenolic compounds in *Opuntia stricta* var. *dillenii* fruit pulp extract (ODP).

Peak ^1^	Compounds	Tr (min)	UV λ_max_ (nm)	MS/MS *m*/*z*
1	Ascorbic acid	3.168	285	115, 89
2	Citric acid	4.083	233	111, 87, 67
3	Quinic acid	5.092	230, 272	111, 85, 67
6	Piscidic acid	8.196	272	193, 165, 135, 119
7	Betanin	10.889	535	390, 389
8	Isobetanin	15.845	535	390, 389
9	Euchomic acid	21.568	278	195, 179, 149, 133
12	2′-O-Apiosyl-4-O-filocactin	27.933	537	551
13	5″-O-E-Sinapoyl-2′-apyosil-filocactin	29.323	540	-
14	Neobetanin	30.633	467	387
15	Neobetanin isomer II	35.343	448	387
17	Neobetanin isomer III	37.034	444	387
19	Isorhamnetin glucoxyl-rhamnosyl-rhamnoside (IG1)	40.529	254, 355	625, 317, 85
20	Isorhamnetin glucoxyl-rhamnosyl-pentoside (IG2)	42.219	250, 353	317, 167, 86

^1^ The numbers are in accordance with the HPLC chromatograms (Figures S2–S5 in the Supplementary Materials).

**Table 2:** Physicochemical characteristics of Brazil nut beverages (BNBs) and Brazil nut beverages with 0.5% (BN ED 0.5%) and 1% (BN ED 1%) of *Opuntia stricta* var. *dillenii* pulp extract during storage at 5 °C for 24 days.

	Sample ^1^	Days of Storage at 5 °C					
		0	1	3	8	12	24
pH	BNB	6.64 ± 0.02 ^Ab^	6.97 ± 0.06 ^Aa^	6.51 ± 0.02 ^Ab^	7.02 ± 0.01 ^Ab^	6.19 ± 0.01 ^Ab^	5.59 ± 0.01 ^Ac^
	BN ED 0.5%	5.22 ± 0.01 ^Bb^	5.80 ± 0.14 ^Ba^	5.37 ± 0.02 ^Bb^	4.97 ± 0.02 ^Bb^	5.16 ± 0.02 ^Bb^	5.15 ± 0.00 ^Bc^
	BN ED 1%	4.77 ± 0.01 ^Cb^	5.24 ± 0.02 ^Ca^	4.90 ± 0.02 ^Cb^	4.80 ± 0.02 ^Cb^	5.04 ± 0.01 ^Cb^	4.50 ± 0.01 ^Cc^
Acidity ^2^	BNB	0.03 ± 0.00 ^Aa^	0.03 ± 0.00 ^Aa^	0.03 ± 0.00 ^Aa^	0.03 ± 0.00 ^Aa^	0.04 ± 0.00 ^Aa^	0.07 ± 0.00 ^Ab^
	BN ED 0.5%	0.07 ± 0.00 ^Ba^	0.08 ± 0.00 ^Ba^	0.08 ± 0.00 ^Ba^	0.08 ± 0.00 ^Ba^	0.08 ± 0.00 ^Ba^	0.09 ± 0.00 ^Bb^
	BN ED 1%	0.15 ± 0.00 ^Ca^	0.13 ± 0.00 ^Ca^	0.13 ± 0.00 ^Ca^	0.12 ± 0.00 ^Ca^	0.13 ± 0.00 ^Ca^	0.15 ± 0.01 ^Cb^
Soluble solid (^o^Brix)	BNB	1.70 ± 0.14 ^a^	1.80 ± 0.14 ^ab^	1.75 ± 0.07 ^ab^	2.00 ± 0.00 ^ab^	1.65 ± 0.07 ^b^	0.20 ± 0.00 ^c^
	BN ED 0.5%	2.20± 0.14 ^a^	2.20± 0.14 ^ab^	2.10± 0.14 ^ab^	2.20± 0.14 ^ab^	1.60 ± 0.14 ^b^	0.40 ± 0.00 ^c^
	BN ED 1%	3.75 ± 0.21 ^a^	2.65 ± 0.21 ^ab^	2.35 ± 0.21 ^ab^	2.45± 0.07 ^ab^	1.70 ± 0.14 ^b^	0.20 ± 0.00 ^c^
L*	BNB	27.55 ± 0.86 ^Aa^	24.01 ± 0.60 ^Aab^	26.41 ± 0.64 ^Aab^	22.73 ± 0.05 ^Aab^	21.52 ± 0.10 ^Aab^	20.22 ± 0.17 ^Ab^
	BN ED 0.5%	2.79 ± 0.95 ^Ba^	2.14 ± 0.01 ^Bab^	2.14 ± 0.02 ^Bab^	2.52 ± 0.10 ^Bab^	2.52 ± 0.03 ^Bab^	2.06 ± 0.06 ^Bb^
	BN ED 1%	1.16 ± 0.04 ^Ca^	1.48 ± 0.17 ^Cab^	1.61 ± 0.03 ^Cab^	1.33 ± 0.03 ^Cab^	0.94 ± 0.18 ^Cab^	1.09 ± 0.04 ^Cb^
a*	BNB	1.08 ± 0.23 ^A^	0.45 ± 0.06 ^A^	0.82 ± 0.02 ^A^	0.69 ± 0.12 ^A^	2.38 ± 0.18 ^A^	2.49 ± 0.40 ^A^
	BN ED 0.5%	11.88 ± 2.69 ^B^	8.60 ± 0.48 ^B^	12.69 ± 0.42 ^B^	12.60 ± 0.23 ^B^	13.01 ± 0.19 ^B^	11.54 ± 0.51 ^B^
	BN ED 1%	5.81 ± 3.63 ^C^	7.81 ± 0.36 ^C^	6.27± 0.13 ^C^	6.06 ± 0.09 ^C^	8.99 ± 1.25 ^C^	6.46 ± 0.39 ^C^
b*	BNB	9.88 ± 0.41 ^Ab^	15.60 ± 0.26 ^Aa^	11.49 ± 0.27 ^Abc^	12.66 ± 0.45 ^Abc^	9.27 ± 1.02 ^Ad^	10.00 ± 0.40 ^Acd^
	BN ED 0.5%	2.46 ± 2.10 ^Ba^	5.68 ± 0.44 ^Ba^	2.14 ± 1.19 ^Bbc^	1.61 ± 1.22 ^Bbc^	0.14 ± 0.08 ^Bd^	1.03 ± 0.36 ^Bcd^
	BN ED 1%	3.06 ± 0.25 ^Ca^	2.78 ± 0.08 ^Ca^	2.14 ± 1.19 ^Cbc^	0.47 ± 0.12 ^Cbc^	4.47 ± 0.12 ^Cd^	4.48 ± 2.11 ^Ccd^
∆E	BNB	-	-	-	-	-	-
	BN ED 0.5%	28.07 ± 1.47 ^a^	25.55 ± 0.06 ^ab^	28.42 ± 0.75 ^a^	25.90 ± 0.14 ^abc^	23.51 ± 0.48 ^bc^	22.36 ± 0.44 ^c^
	BN ED 1%	27.84 ± 0.44 ^a^	26.73 ± 0.38 ^ab^	26.90 ± 0.34 ^a^	25.18 ± 0.11 ^abc^	25.13 ± 2.27 ^bc^	22.14 ± 2.80 ^c^

^1^ The results are provided as means ± standard deviations. Different uppercase letters indicate statistically significant differences in a column. Different lowercase letters indicate statistically significant differences between the days of conservation in the same sample (*p* < 0.05). ^2^ Expressed as g of citric acid/100 mL.

**Table 3:** Particle size and zeta potential of Brazil nut beverages (BNBs) and Brazil nut beverages with added 0.5% (BN ED 0.5%) and 1% (BN ED 1%) of *Opuntia stricta* var. *dillenii* pulp extract during storage at 5 °C for 24 days.

Sample	Particle Size (nm) ^1^					
	Day 0	Day 1	Day 3	Day 8	Day 12	Day 24
BNB	861 ± 80 ^Aa^	2171 ± 221 ^Aab^	2701 ± 196 ^Abc^	2991 ± 180 ^Ac^	5527 ± 241 ^Ad^	7554 ± 263 ^Ad^
BN ED 0.5%	3583 ± 233 ^Ba^	3975 ± 83 ^Bab^	4577 ± 177 ^Bbc^	5429 ± 118 ^Bc^	6228 ± 410 ^Bd^	8073 ± 286 ^Bd^
BN ED 1%	5969 ± 535 ^Ca^	7584 ± 209 ^Cab^	8785 ± 261 ^Cbc^	9168 ± 159 ^Cc^	10,302 ± 129 ^Cd^	9110 ± 628 ^Cd^
Zeta potential (mV) ^1^						
BNB	42.34 ± 0.36 ^Aa^	34.45 ± 2.14 ^Ab^	33.96 ± 1.38 ^Abc^	31.72 ± 0.48 ^Abc^	27.71 ± 0.76 ^Ac^	21.53 ± 0.93 ^Ad^
BN ED 0.5%	27.22 ± 0.25 ^Ba^	24.46 ± 3.10 ^Bb^	24.11 ± 1.31 ^Bbc^	17.99 ± 1.64 ^Bbc^	18.89 ± 0.81 ^Bc^	16.66 ± 1.98 ^Bd^
BN ED 1%	19.31 ± 2.94 ^Ca^	16.53 ± 0.91 ^Cb^	16.07 ± 1.89 ^Cbc^	17.41 ± 1.23 ^Cbc^	16.26 ± 2.17 ^Cc^	10.26 ± 2.42 ^Cb^

^1^ Data are provided as means and standard deviations (*n* = 3). Different uppercase letters indicate statistically significant differences (*p* < 0.05) among samples at the same storage time. Different lowercase letters indicate significant differences (*p* < 0.5) in the storage time for the same sample. BNB: Brazil nut beverage; BN ED 0.5%: Brazil nut beverage with 0.5% OPD extract; and NB ED 1%: Brazil nut beverage with 1% OPD extract.

**Table 4:** Characterization of main individual betalain and phenolic compounds of Brazil nut beverages with added *Opuntia stricta* var. *dillenii* fruit pulp extract (OPD).

Peak	Compounds ^1^	Sample	tR (min)	UV λ_max_ (nm)	MS/MS *m*/*z*
1	Ascorbic acid	BN ED 0.5%	3.20	285	147.2, 87.00, 69.03
		BN ED 1%	3.20	285	
4	Succinic acid	BN ED 0.5%	6.13	228	72.91
		BN ED 1%	6.13	228	
5	Gallic acid	BN ED 0.5%	6.71	274	125.02, 107.01, 97.03, 79.02, 69.03, 51.02, 41.04
		BN ED 1%	6.70	274	
6	Piscidic acid	BN ED 0.5%	8.19	272	193, 165, 135, 119
		BN ED 1%	8.19	272	
7	Betanin	BN ED 0.5%	11.00	535	390, 389
		BN ED 1%	11.02	535	
8	Isobetanin	BN ED 0.5%	15.95	535	390, 389
		BN ED 1%	15.98	535	
9	Euchomic acid	BN ED 0.5%	21.89	278	195, 179, 149, 133
		BN ED 1%	21.89	278	
10	Catechin	BN ED 0.5%	23.64	230, 280	136.8, 150.7, 160.8
		BN ED 1%	23.63	230, 280	
11	Catechin derivative	BN ED 0.5%	26.76	282	289.1
		BN ED 1%	26.76	282	
14	Neobetanin	BN ED 0.5%	30.81	467	387
		BN ED 1%	30.79	467	
15	Neobetanin isomer II	BN ED 0.5%	35.34	448	387
		BN ED 1%	35.32	448	
16	P-coumaric acid	BN ED 0.5%	36.09	228, 310	119.05, 91.05
		BN ED 1%	36.09	228, 310	
17	Neobetanin isomer III	BN ED 0.5%	37.91	444	387
		BN ED 1%	37.93	444	
18	Taxofolin (dihydroquercitin)	BN ED 0.5%	38.65	233, 312	285.05, 179.00, 125.03
		BN ED 1%	38.65	233, 312	
19	Isorhamnetin glucoxyl-rhamnosyl-rhamnoside (IG1)	BN ED 0.5%	40.26	254, 355	625, 317, 85
		BN ED 1%	40.32	254, 355	
20	Isorhamnetin glucoxyl-rhamnosyl-pentoside (IG2)	BN ED 0.5%	42.21	250, 353	317, 167, 86
		BN ED 1%	42.23	250, 353	

^1^ Compounds in capital letters were only present in Brazil nut beverage without OPD extract.

**Table 5:** Content of the main individual betalains and phenolic compounds present in the pulp of *Opuntia stricta* var. *dillenii* extract (control) and Brazil nut beverages with 0.5% (BN ED 0.5) and 1% (BN ED 1%) of added *Opuntia stricta dillenii* pulp extract at 5 °C for 24 days.

Compound	ODP Extract (µg/gr Extract) ^3^	Beverage Sample ^2^	Content (µg/g BN Beverage) ^1^					
			Days of Storage at 5 °C					
			0	1	3	8	12	24
Piscidic acid	4262.18 ± 77.70	BN ED 1%	161.75 ± 0.71 ^Ba^	158.21 ± 0.70 ^Bab^	153.24 ± 0.68 ^Bab^	152.18 ± 0.67 ^Bb^	138.34 ± 0.61 ^Bc^	135.50 ± 0.60 ^Bc^
		BN ED 0.5%	70.11 ± 0.35 ^Aa^	68.30 ± 0.63 ^Aab^	63.66 ± 0.72 ^Aab^	60.33 ± 0.51 ^Ab^	59.10 ± 0.67 ^Ac^	55.78 ± 0.76 ^Ac^
Betanin		BN ED 1%	326.21 ± 3.55 ^Ba^	301.89 ± 2.24 ^Bb^	276.47 ± 2.05 ^Babc^	271.89 ± 2.01 ^Babc^	258.19 ± 1.91 ^Bbc^	247.71 ± 1.84 ^Bc^
	8188.12 ± 88.94	BN ED 0.5%	124.63 ± 0.92 ^Aa^	124.00 ± 0.92 ^Ab^	121.46 ± 0.90 ^Aabc^	118.13 ± 0.88 ^Aabc^	115.06 ± 0.85 ^Abc^	114.10 ± 0.85 ^Ac^
Isobetanin		BN ED 1%	188.93 ± 2.30 ^Ba^	171.50 ± 2.10 ^Bb^	155.76 ± 1.10 ^Bbc^	154.10 ± 1.88 ^Bbc^	144.27 ± 1.75 ^Bbc^	142.94 ± 1.00 ^Bc^
	5042.88 ± 101.86	BN ED 0.5%	74.92 ± 0.53 ^Aa^	72.85 ± 0.51 ^Ab^	69.94 ± 0.50 ^Abc^	68.86 ± 0.48 ^Abc^	68.24 ± 0.48 ^Abc^	64.01 ± 0.45 ^Ac^
Neobetanin		BN ED 1%	23.26 ± 0.45 ^Ba^	21.85 ± 0.42 ^Ba^	20.41 ± 0.39 ^Bab^	19.93 ± 0.38 ^Bab^	17.16 ± 0.33 ^Bbc^	16.68 ± 0.32 ^Bc^
	4039.62 ± 55.88	BN ED 0.5%	6.51 ± 0.13 ^Aa^	6.23 ± 0.12 ^Aa^	5.87 ± 0.12 ^Aab^	5.54 ± 0.11 ^Aab^	5.02 ± 0.10 ^Abc^	3.73 ± 0.07 ^Ac^
Isorhamnetin glucoxyl-rhamnosyl-rhamnoside (IG1)		BN ED 1%	0.64 ± 0.02 ^Ba^	0.62 ± 0.01 ^Bab^	0.60 ± 0.01 ^Bab^	0.55 ± 0.01 ^Bbc^	0.52 ± 0.01 ^Bcd^	0.45 ± 0.01 ^Bd^
	17.46 ± 0.16	BN ED 0.5%	0.29 ± 0.01 ^Aa^	0.25 ± 0.01 ^Aab^	0.23 ± 0.00 ^Aab^	0.23 ± 0.01 ^Abc^	0.20 ± 0.00 ^Acd^	0.20 ± 0.00 ^Ad^
Isorhamnetin glucoxyl-rhamnosyl-pentoside (IG2)		BN ED 1%	1.09 ± 0.03 ^Ba^	1.09 ± 0.03 ^Ba^	1.03 ± 0.05 ^Ba^	1.02 ± 0.05 ^Ba^	0.73 ± 0.01 ^Bab^	0.54 ± 0.04 ^Bb^
	27.59 ± 1.36	BN ED 0.5%	0.54 ± 0.01 ^Aa^	0.52 ± 0.00 ^Aa^	0.51 ± 0.03 ^Aa^	0.50 ± 0.00 ^Aa^	0.43 ± 0.01 ^Aab^	0.43 ± 0.01 ^Ab^
Total betalains	17,270.62	BN ED 1% BN ED 0.5%	538.4 206.06	495.24 203.08	452.64 197.27	445.92 192.53	419.62 188.32	407.33 181.84
Total phenolic	4307.23	BN ED 1% BN ED 0.5%	163.48 70.94	159.92 69.07	154.87 64.4	153.76 61.06	139.59 59.73	136.49 56.41

^1^ Data are provided as means and standard deviations (*n* = 3). Different uppercase letters indicate statistically significant differences (*p* < 0.05) among samples at the same storage time. Different lowercase letters indicate significant differences (*p* < 0.5) in storage times for the same sample. ^2^ BN ED 0.5%: Brazil nut beverage with 0.5% OPD extract; NB ED 1%: Brazil nut beverage with 1% ODP extract. ^3^ The control corresponds to extract from the pulp of *Opuntia stricta* var. *dillenii* (ODP).

## Data Availability

The original contributions presented in the study are included in the article/Supplementary Materials, further inquiries can be directed to the corresponding author.

## Supplementary Materials

The following supporting information can be downloaded at https://www.mdpi.com/article/10.3390/foods13081237/s1: Figure S1: Image of Brazil nut (*Bertholletia excels* HBK); Figure S2: HPLC chromatogram of betalains and phenolic compounds from (a) OPD, (b) BN ED 1%, and (c) BN ED 0.5% at 280 nm in which the numbers correspond to the identified compounds shown in Table 1 and Table 4; Figure S3: HPLC chromatogram of betalains and phenolic compounds from (a) OPD, (b) BN ED 1%, and (c) BN ED 0.5% at 370 nm in which the numbers correspond to the identified compounds shown in Table 1 and Table 4; Figure S4: HPLC chromatogram of betalains and phenolic compounds from (a) OPD, (b) BN ED 1%, and (c) BN ED 0.5% at 480 nm in which the numbers correspond to the identified compounds shown in Table 1 and Table 4; Figure S5: HPLC chromatogram of betalains and phenolic compounds from (a) OPD, (b) BN ED 1%, and (c) BN ED 0.5% at 535 nm in which the numbers correspond to the identified compounds shown in Table 1 and Table 4; Table S1: Physicochemical characteristics of Brazil nuts (BNs), fresh *Opuntia stricta* var. *dillenii* fruits, and standardized Brazil nut beverage (BNB); Table S2: Encapsulation efficiency of main betalains and phenolic compounds of Brazil nut beverages with 0.5% (BN ED 0.5%) and 1% (BN ED 1%) *Opuntia stricta* var. *dillenii* pulp extract added during cold storage at 5 °C for 24 days; Table S3: Total phenolic content (TPC) and oxygen radical absorbance capacity (ORAC) of Brazil nut beverages (BNs) and BN beverages with 0.5% (BN ED 0.5%) and 1% (BN ED 1%) of *Opuntia stricta* var. *dillenii* pulp extract added during storage at 5 °C for 24 days analyzed after TCA extraction method; and Table S4: Total phenolic content (TPC) and oxygen radical absorbance capacity (ORAC) of Brazil nut beverages (BNs) and BN beverages with 0.5% (BN ED 0.5%) and 1% (BN ED 1%) of *Opuntia stricta* var. *dillenii* pulp extract added during storage at 5 °C for 24 days directly analyzed from the beverages.
