# Supplementary material for: A Healthy Brazil Nut Beverage with Opuntia stricta var. dillenii Green Extract: Beverage Stability and Changes in Bioactives and Antioxidant Activity during Cold Storage
Source: Foods. 2024 Apr 18;13(8):1237. doi: 10.3390/foods13081237 (PMC11049168; doi:10.3390/foods13081237)
Supplement: Supplementary file 1 [file foods-13-01237-s001.zip › foods-2936653-supplementary.pdf]

foods

SUPLEMENTARY MATERIAL

Figure S1. Image of Brazil nut (*Bertholletia excels* HBK)

Figure S2. HPLC chromatogram of betalains and phenolic compounds from (**a**) OPD, (**b**) BN ED 1%, and (**c**) BN ED 0.5% at 280 nm. Numbers correspond to the identified compounds shown in Tables 1 and 4.

Figure S3. HPLC chromatogram of betalains and phenolic compounds from (**a**) OPD, (**b**) BN ED 1%, and (**c**) BN ED 0.5% at 370 nm. Numbers correspond to the identified compounds shown in Tables 1 and 4.

Figure S4. HPLC chromatogram of betalains and phenolic compounds from (**a**) OPD, (**b**) BN ED 1%, and (**c**) BN ED 0.5% at 480 nm. Numbers correspond to the identified compounds shown in Tables 1 and 4.

Figure S5. HPLC chromatogram of betalains and phenolic compounds from (**a**) OPD, (**b**) BN ED 1%, and (**c**) BN ED 0.5% at 535 nm. Numbers correspond to the identified compounds shown in Tables 1 and 4.

Table S1. Physicochemical characteristics of Brazil nuts (BN), fresh *Opuntia stricta* var. *dillenii* fruits, and standardized Brazil nut beverage (BNB).

Table S2. Encapsulation efficiency of main betalains and phenolic compounds of Brazil nut beverages with 0.5% (BN ED 0.5%) and 1% (BN ED 1%) Opuntia stricta dillenii pulp extract added during cold storage at 5 ºC for 24 days.

Table S3. Total phenolic content (TPC) and oxygen radical absorbance capacity (ORAC) of Brazil nut beverages (BNs) and BN beverages with 0.5% (BN ED 0.5%) and 1% (BN ED 1%) of Opuntia stricta dillenii pulp extract added during storage at 5°C for 24 days analyzed after TCA extraction method.

Table S4. Total phenolic content (TPC) and oxygen radical absorbance capacity (ORAC) of Brazil nut beverages (BNs) and BN beverages with 0.5% (BN ED 0.5%) and 1% (BN ED 1%) of Opuntia Stricta var. Dillenii pulp extract added during storage at 5°C for 24 days directly analyzed from the beverages.

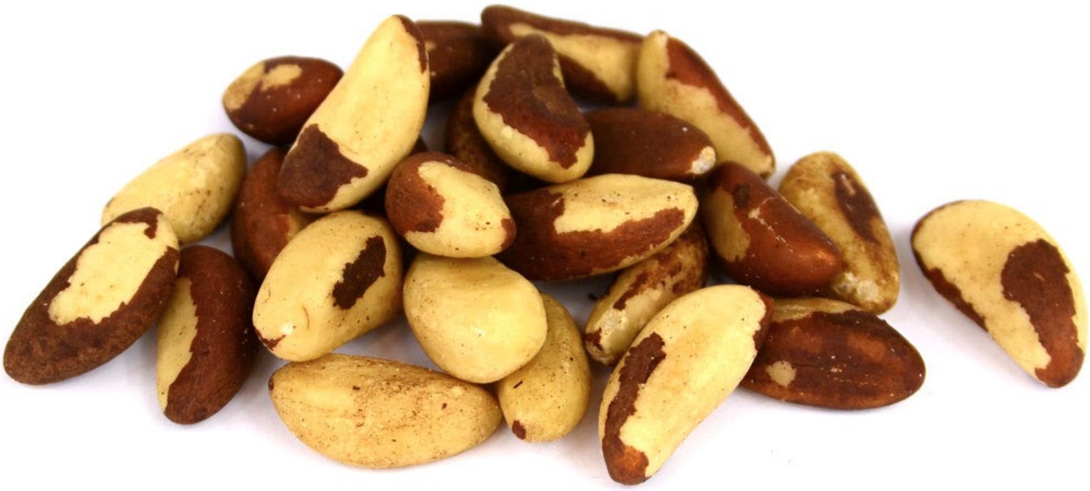

Figure S1. Image of Brazil nut (*Bertholletia excels* HBK).

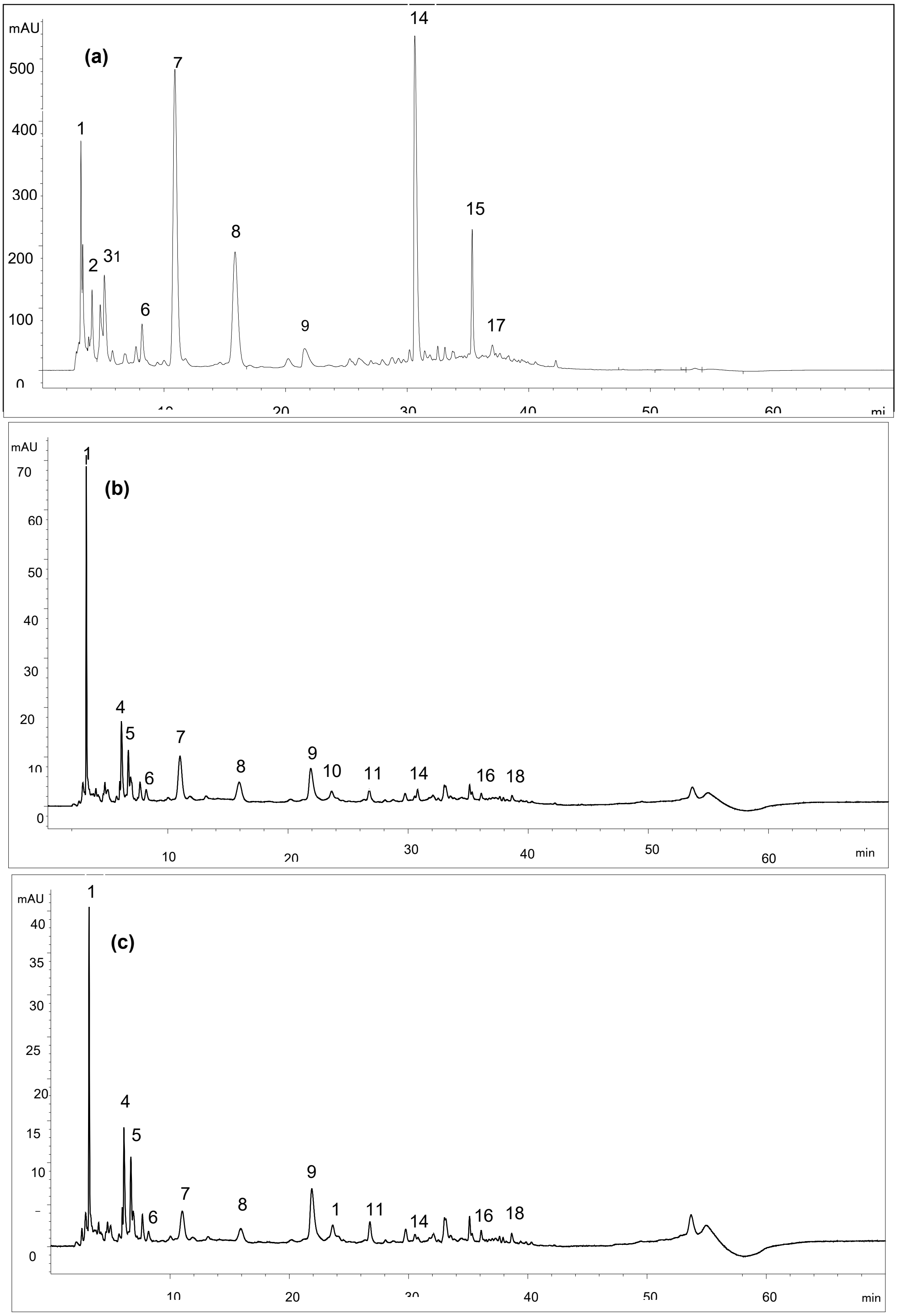

Figure S2. HPLC chromatogram of betalains and phenolic compounds from (**a**) OPD, (**b**) BN ED 1%, and (**c**) BN ED 0.5% at 280 nm. Numbers correspond to the identified compounds shown in Tables 1 and 4.

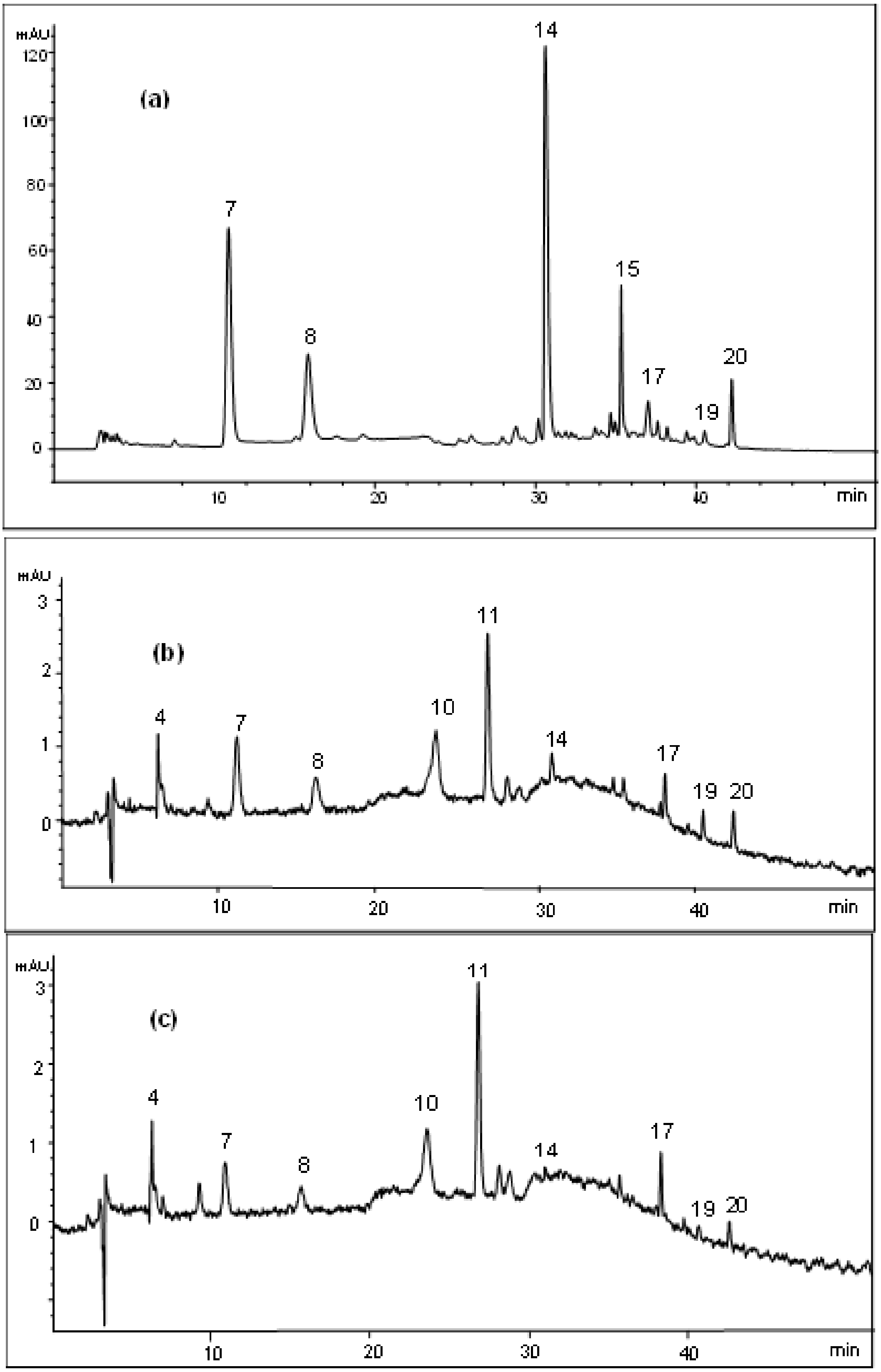

Figure S3. HPLC chromatogram of betalains and phenolic compounds from (**a**) OPD, (**b**) BN ED 1%, and (**c**) BN ED 0.5% at 370 nm. Numbers correspond to the identified compounds indicated in Tables 4 and 5.

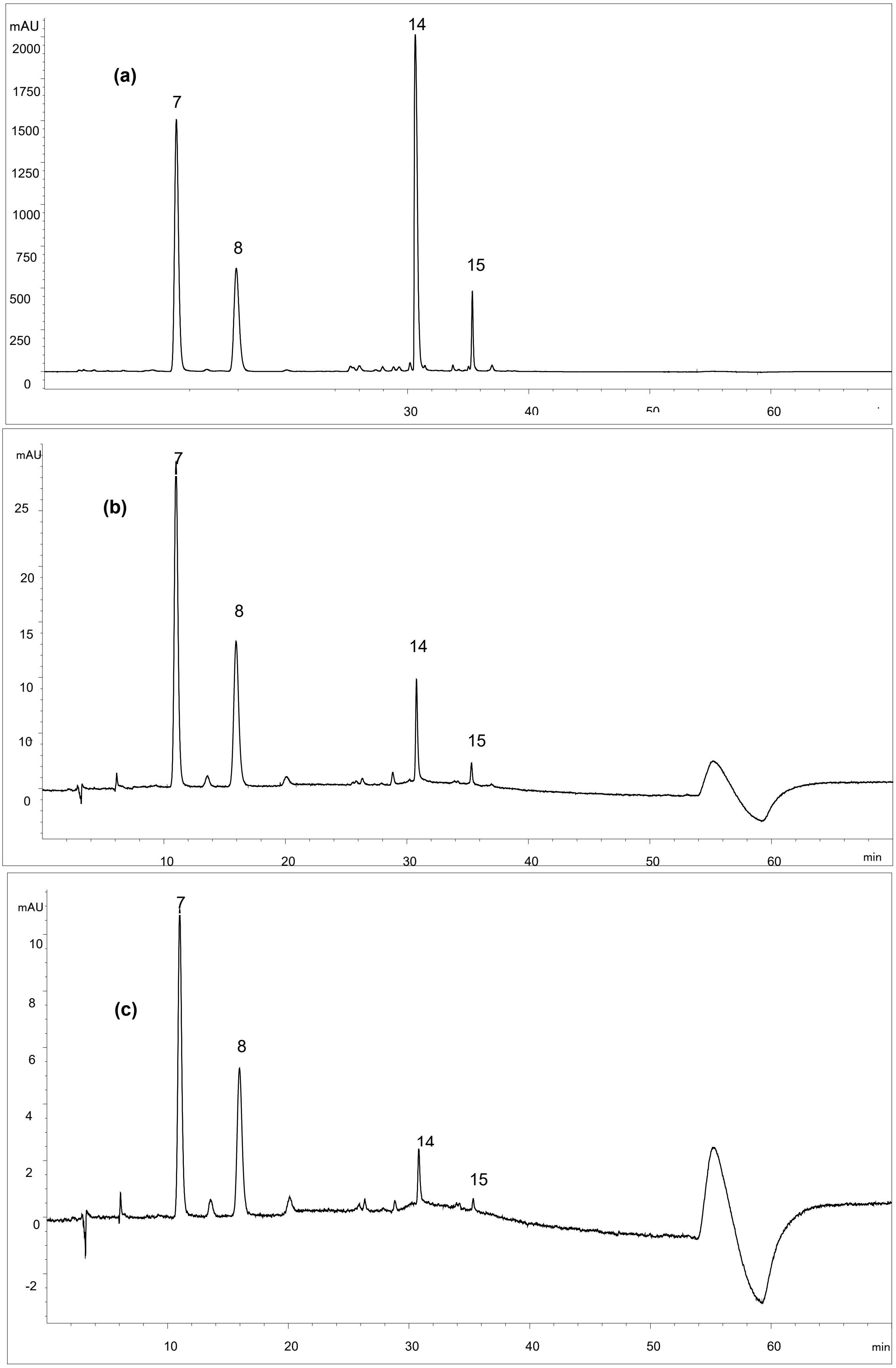

Figure S4. HPLC chromatogram of betalains and phenolic compounds from (**a**) OPD, (**b**) BN ED 1%, and (**c**) BN ED 0.5% at 480 nm. Numbers correspond to the identified compounds shown in Tables 1 and 4.

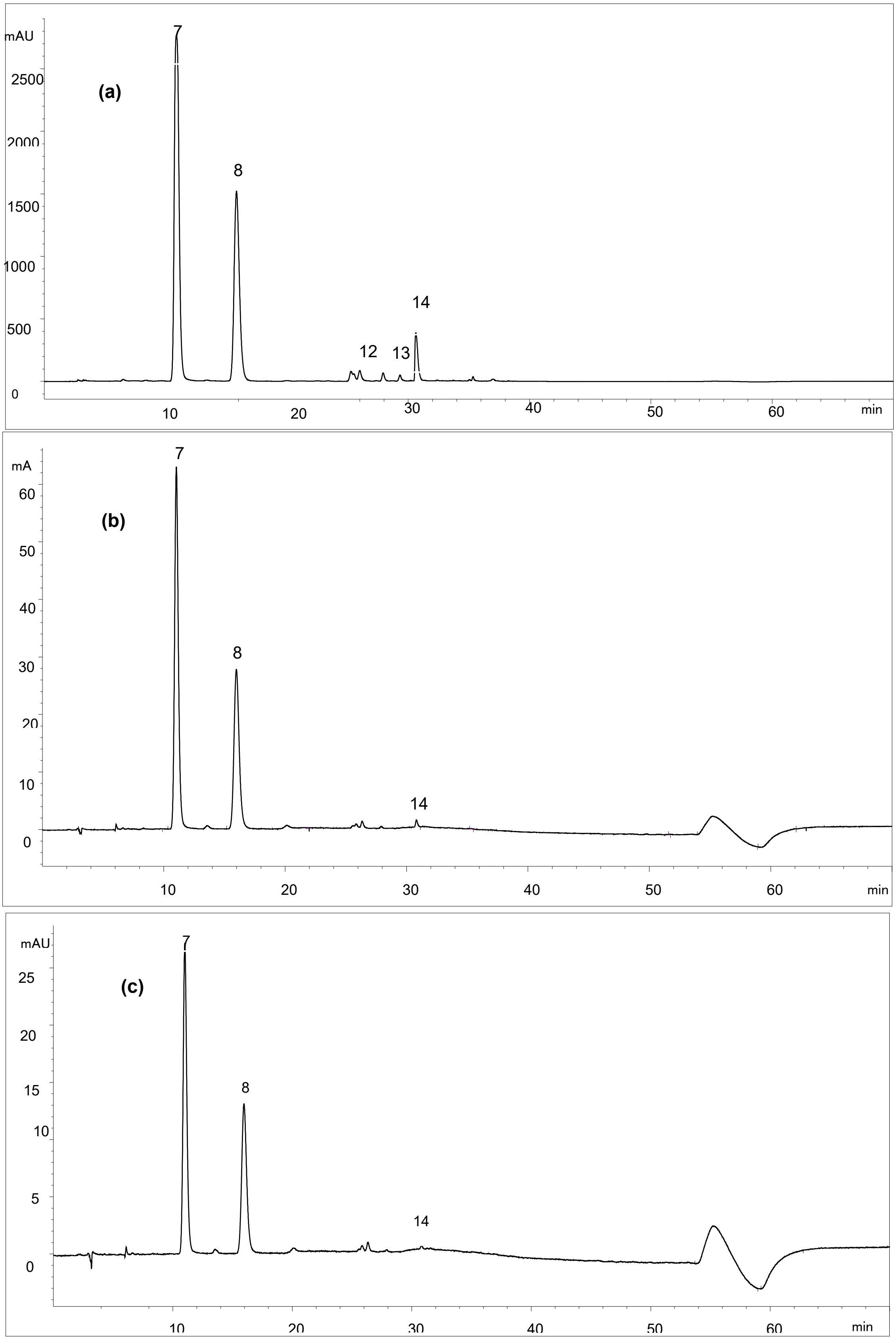

Figure S5. HPLC chromatogram of betalains and phenolic compounds from (**a**) OPD, (**b**) BN ED 1%, and (**c**) BN ED 0.5% at 535 nm. Numbers correspond to the identified compounds shown in Tables 1 and 4.

Table S1. Physicochemical characteristics of Brazil nuts (BNs), fresh *Opuntia stricta* var. *dillenii* fruits, and standardized Brazil nut beverage (BNB).

Characteristic	Brazil Nut (BN)	*Opuntia stricta var. dillenii* Fruit Pulp	Brazil Nut Standardized Beverage (BNB)
Protein *(%, ω/ω)*	17.3 ± 0.4	-	1.27 ± 0.0
Lipid *(%, ω/ω)*	66.1 ± 1.1	-	2.9 ± 0.0
Carbohydrates[1] *(%, ω/ω)*	10.9 ± 1.5	36.7 ± 5.7	1.06 ± 1.0
Ashes *(%, ω/ω)*	3.4 ± 0.02	-	0.14 ± 0.0
Energy (kcal)	707.9 ± 1.5	-	41.3 ± 1.1
Total solids *(%, ω/ω)*	97.8 ± 0.03	17.55 ± 2.82	5.7 ± 0.3
Wet *(%, ω/ω)*	2.2 ± 0.03	82.45 ± 3.21	94.3 ± 0.3
Ph	6.14 ± 0.1	3.28 ± 0.05	6.65 ± 0.1
Acidity[2]	0.18 ± 0.0	1.58 ± 0.1	0.03 ± 0.0
Soluble solids (ºBrix)	14.5 ± 0.1	11.6 ± 0.2	2.23 ± 0.1

[1] Total carbohydrates of fruit pulp in g of carbohydrates/100 g of *O. dillenii* pulp. [2] Expressed as g of citric acid/100 ml of beverage or g of citric acid/100 g of *O. dillenii* fruit pulp or Brazil nut.

Table S2. Encapsulation efficiency of main betalains and phenolic compounds of Brazil nut beverages with 0.5% (BN ED 0.5%) and 1 % (BN ED 1%) Opuntia stricta dillenii pulp extract added during cold storage at 5 °C for 24 days.

Recovery Efficiency (%)							
		Days of Storage at 5 °C					
**COMPOUND**	**Samples**	**0**	**1**	**3**	**8**	**12**	**24**
**Piscidic acid**	**BN ED 1%**	93.67 ± 0.41 ^{Ba}	91.61 ± 0.41 ^{Ba}	88.74 ± 0.39 ^{Bb}	88.12 ± 0.39 ^{Bb}	80.11 ± 0.35 ^{Bc}	78.47 ± 0.35 ^{Bc}
	**BN ED 0.5%**	81.20 ± 0.40 ^{Aa}	79.11 ± 0.73 ^{Aa}	73.73 ± 0.83 ^{Ab}	69.87 ± 0.59 ^{Ab}	68.45 ± 0.77 ^{Ac}	64.60 ± 0.88 ^{Ac}
**Betanin**	**BN ED 1%**	98.84 ± 1.07 ^{Ba}	91.47 ± 0.68 ^{Bab}	83.77 ± 0.62 ^{Bbc}	82.38 ± 0.61 ^{Bbc}	78.23 ± 0.58 ^{Bc}	75.06 ± 0.56 ^{Bc}
	**BN ED 0.5%**	75.53 ± 0.56 ^{Aa}	75.15 ± 0.56 ^{Aab}	73.61 ± 0.54 ^{Abc}	71.59 ± 0.53 ^{Abc}	69.73 ± 0.52 ^{Ac}	69.15 ± 0.52 ^{Ac}
**Isobetanin**	**BN ED 1%**	92.35 ± 1.13 ^{Ba}	83.82 ± 1.02 ^{Bb}	76.13 ± 0.54 ^{Bc}	75.32 ± 0.92 ^{Bc}	70.51± 0.86 ^{Bcd}	69.87 ± 0.49 ^{Bd}
	**BN ED 0.5%**	73.24 ± 0.52 ^{Aa}	71.22 ± 0.50 ^{Ab}	68.37 ± 0.49 ^{Ac}	67.32 ± 0.47 ^{Ac}	66.71 ± 0.47 ^{Acd}	62.57 ± 0.44 ^{Ad}
**Neobetanin**	**BN ED 1%**	14.26 ± 0.27 ^{Ba}	13.39 ± 0.26 ^{Bab}	12.51 ± 0.24 ^{Bbc}	12.22 ± 0.24 ^{Bc}	10.52 ± 0.20 ^{Bd}	10.22 ± 0.20 ^{Be}
	**BN ED 0.5%**	7.98 ± 0.16 ^{Aa}	7.64 ± 0.15 ^{Aab}	7.20 ± 0.14 ^{Abc}	6.79 ± 0.13 ^{Ac}	6.16 ± 0.12 ^{Ad}	4.57 ± 0.08 ^{Ae}
**Isorhamnetin glucoxyl-rhamnosyl-rhamnoside (IG1)**	**BN ED 1%**	91.56 ± 2.24 ^{Ba}	87,66 ± 0.69 ^{Bb}	85.71 ± 0.77 ^{Bbc}	78.84 ± 1.93 ^{Bc}	73.76 ± 1.80 ^{Bd}	63.58 ± 1.55 ^{Bd}
	**BN ED 0.5%**	82.94 ± 2.03 ^{Aa}	71.21 ± 1.74 ^{Ab}	66.23 ± 0.59 ^{Abc}	66.13 ± 1.62 ^{Ac}	55.96 ± 1.36 ^{Ad}	55.96 ± 1.36 ^{Ad}
**Isorhamnetin glucoxyl-rhamnosyl-pentoside (IG2)**	**BN ED 1%**	95.40 ± 2.87 ^{a}	95.07 ± 2.41 ^{a}	90.22 ± 4.46 ^{a}	89.00 ± 4.40 ^{a}	63.81 ± 1.08 ^{b}	46.45 ± 1.08 ^{b}
	**BN ED 0.5%**	93.70 ± 2.38 ^{a}	90.74 ± 0.64 ^{a}	89.25 ± 4.76 ^{a}	87.37 ± 0.44 ^{a}	74.75 ± 1.85 ^{b}	74.75 ± 1.85 ^{b}

[1] Data are provided as means and standard deviations (n=3). Different uppercase letters indicate statistically significant differences ($p < 0.05$) among samples at the same storage time. Different lowercase letters indicate significant differences ($p < 0.5$) in storage times for the same sample. [2] BN ED 0.5%: Brazil nut beverage with 0.5% OPD extract; NB ED 1%: Brazil nut beverage with 1% OPD extract.

Table S3. Total phenolic content (TPC) and oxygen radical absorbance capacity (ORAC) of Brazil nut beverages (BNs) and BN beverages with 0.5% (BN ED 0.5%) and 1% (BN ED 1%) of Opuntia stricta dillenii pulp extract added during storage at 5 °C for 24 days analyzed after TCA extraction method.[3.]

**TPC (mg of GAE/100 mL of BN Beverage) [1]**						
			**Days of Storage at 5 °C**			
**Sample [2]**	**0**	**1**	**3**	**8**	**12**	**24**
**BNB**	13.57 ± 0.02 Aa	12.5 ± 0.21 Aa	11.91 ± 0.06 Ab	11.50 ± 0.09 Abc	10.41 ± 0.1 Acd	10.18 ± 0.08 Ad
**BN ED 0.5%**	42.23 ± 0.02 Ba	41.41 ± 0.74 Ba	39.18 ± 0.03 Bb	38.45 ± 0.08 Bbc	38.16 ± 0.02 Bcd	37.61 ± 0.05 Bd
**BN ED 1%**	63.61 ± 0.14 Ca	62.85 ± 0.01 Ca	60.60 ± 0.67 Cb	60.94 ± 0.06 Cbc	59.23 ± 0.30 Ccd	57.03 ± 0.04 Cd
**ORAC (µmol of TE/g BN of Beverage) [1]**						
**BNB**	0.72 ± 0.04 Aa	0.70 ± 0.04 Aab	0.66 ± 0.06 Abc	0.64 ± 0.05 Acd	0.53 ± 0.04 Ade	0.49 ± 0.04 Ae
**BN ED 0.5%**	1.80 ± 0.05 Ba	1.76 ± 0.04 Bab	1.71 ± 0.03 Bbc	1.66 ± 0.04 Bcd	1.63 ± 0.01 Bde	1.61 ± 0.04 Be
**BN ED 1%**	3.67 ± 0.05 Ca	3.62 ± 0.03 Cab	3.56 ± 0.05 Cbc	3.52 ± 0.08 Ccd	3.45 ± 0.08 Cde	3.37 ± 0.09 Ce

[1] Data are provided as means and standard deviations (n = 3). Different uppercase letters indicate statistically significant differences ($p < 0.05$) among samples at the same storage time. Different lowercase letters indicate significant differences ($p < 0.5$) in storage times for the same sample. [2] BN ED 0.5%: Brazil nut beverage with 0.5% OPD extract; NB ED 1%: Brazil nut beverage with 1% OPD extract. [3] TCA extraction method reported by Naderi et al. (2010).

Table S4. Total phenolic content (TPC) and oxygen radical absorbance capacity (ORAC) of Brazil nut beverages (BNs) and BN beverages with 0.5% (BN ED 0.5%) and 1% (BN ED 1%) of Opuntia Stricta var. Dillenii pulp extract added during storage at 5 °C for 24 days directly analyzed from the beverages.[1.]

**TPC (mg of GAE/100 mL of Beverage) [2]**						
			**Days of Storage at 5 °C**			
**Sample [3]**	**0**	**1**	**3**	**8**	**12**	**24**
**BNB**	11.38 ± 1.66 Aa	11 ± 1.44 Aa	9.69 ± 0.27 Aab	8.27 ± 0.07 Abc	10.41 ± 0.55 Acd	6.25 ± 0.35 Ad
**BN ED 0.5%**	35.67 ± 0.64 Ba	33.38 ± 1.24 Ba	32.15 ± 1.21 Bab	31.3 ± 1.51 Bbc	29.19 ± 1.34 Bcd	25.29 ± 1.18 Bd
**BN ED 1%**	53.18 ± 1.51 Ca	51.18 ± 1.94 Ca	49.95 ± 1.8 Cab	45.45 ± 2.98 Cbc	45.45 ± 3.15 Ccd	40.18 ± 2.76 Cd
**ORAC (µmol of TE/gr of Beverage) [1]**						
**BNB**	0.50 ± 0.03 Aa	0.48 ± 0.03 Aab	0.46 ± 0.01 Aab	0.41 ± 0.01 Abc	0.38 ± 0.03 Acd	0.33 ± 0.04 Ad
**BN ED 0.5%**	1.58 ± 0.06 Ba	1.56 ± 0.07 Bab	1.50 ± 0.04 Bab	1.66 ± 0.04 Bbc	1.46 ± 0.06 Bcd	1.39 ± 0.06 Bd
**BN ED 1%**	2.98 ± 0.06 Ca	2.94 ± 0.06 Cab	2.91 ± 0.04 Cab	2.87 ± 0.04 Cbc	2.81 ± 0.02 Ccd	2.75 ± 0.04 Cd

[1] Total phenolic content (TPC) and oxygen radical absorbance capacity (ORAC) analysis was conducted directly from samples without previous extraction. [2] Data are provided as means and standard deviations (n = 3). Different uppercase letters indicate statistically significant differences ($p < 0.05$) among samples at the same storage time. Different lowercase letters indicate significant differences ($p < 0.5$) in storage times for the same sample. [3] BN ED 0.5%: Brazil nut beverage with 0.5% OPD extract; NB ED 1%: Brazil nut beverage with 1% OPD extract.
